# Circadian clock genes promote glioma progression by affecting tumour immune infiltration and tumour cell proliferation

**DOI:** 10.1111/cpr.12988

**Published:** 2021-01-13

**Authors:** Zeyu Wang, Guanhua Su, Ziyu Dai, Ming Meng, Hao Zhang, Fan Fan, Zhengzheng Liu, Longbo Zhang, Nathaniel Weygant, Fengqiong He, Ning Fang, Liyang Zhang, Quan Cheng

**Affiliations:** ^1^ Department of Neurosurgery Xiangya Hospital Central South University Changsha China; ^2^ Clinic Medicine of 5‐year Program Xiangya School of Medicine Central South University Changsha China; ^3^ Department of Oncology Xiangya Hospital Central South University Changsha China; ^4^ Department of Neurosurgery Yale School of Medicine New Haven CT USA; ^5^ Academy of Integrative Medicine Fujian University of Traditional Chinese Medicine Fuzhou China; ^6^ Fujian Key Laboratory of Integrative Medicine in Geriatrics Fujian University of Traditional Chinese Medicine Fujian China; ^7^ Clinical Diagnosis and Therapy Center for Glioma of Xiangya Hospital Central South University Changsha China; ^8^ Department of Gastroenterology The Third Xiangya Hospital Central South University Changsha China; ^9^ National Clinical Research Center for Geriatric Disorders Changsha China; ^10^ Department of Clinical Pharmacology Xiangya Hospital Central South University Changsha China

**Keywords:** cell cycle, circadian clock genes, glioma, immune infiltration, nomogram

## Abstract

**Objectives:**

Circadian rhythm controls complicated physiological activities in organisms. Circadian clock genes have been related to tumour progression, but its role in glioma is unknown. Therefore, we explored the relationship between dysregulated circadian clock genes and glioma progression.

**Materials and Methods:**

Samples were divided into different groups based on circadian clock gene expression in training dataset (n = 672) and we verified the results in other four validating datasets (n = 1570). The GO and GSEA enrichment analysis were conducted to explore potential mechanism of how circadian clock genes affected glioma progression. The single‐cell RNA‐Seq analysis was conducted to verified previous results. The immune landscape was evaluated by the ssGSEA and CIBERSORT algorithm. Cell proliferation and viability were confirmed by the CCK8 assay, colony‐forming assay and flow cytometry.

**Results:**

The cluster and risk model based on circadian clock gene expression can predict survival outcome. Samples were scoring by the least absolute shrinkage and selection operator regression analysis, and high scoring tumour was associated with worse survival outcome. Samples in high‐risk group manifested higher activation of immune pathway and cell cycle. Tumour immune landscape suggested high‐risk tumour infiltrated more immunocytes and more sensitivity to immunotherapy. Interfering TIMELESS expression affected circadian clock gene expression, inhibited tumour cell proliferation and arrested cell cycle at the G0/G1 phase.

**Conclusions:**

Dysregulated circadian clock gene expression can affect glioma progression by affecting tumour immune landscape and cell cycle. The risk model can predict glioma survival outcome, and this model can also be applied to pan‐cancer.

## Graphical abstract

Dysregulated circadian clock genes were associated with glioma grades and the IDH status. Prognostic model suggests circadian clock genes affect glioma progression. The GO and GSEA enrichment analysis suggested dysregulated circadian clock genes can affect glioma through interfering cell cycle and influencing immunocytes infltration.

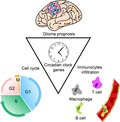

## 1 INTRODUCTION

Glioma is the most common primary intracranial tumour, accounting for 28% of all and 80% of malignant brain tumours, according to the Central Brain Tumor Registry of the United States.^1^ Glioblastoma (GBM, WHO grade IV) is the most malignant glioma with an overall 5‐year survival rate less than 5%.^2^ Clinically, apart from the histologic subtype, other biomarkers including IDH status, 1p19q status and MGMT methylation status are used to predict patient survival and personalize therapy. Lower grade glioma (LGG), IDH mutation, MGMT methylation and 1p19q codeletion are associated with better survival. Current standard treatments include maximal surgical resection along with postoperative radiation and temozolomide concurrent chemotherapy.^3^, ^4^, ^5^ However, the prediction of patients’ survival outcome is still imprecise and the development of an effective prognostic model is in urgency.

Circadian rhythm is an endogenous oscillation of numerous physiological activities in our bodies corresponding to 24‐hour periodicity.^6^, ^7^ Genes involved in circadian rhythm can be divided into two groups, the core clock genes and the clock control genes. The former governs circadian rhythm and consists of ARNTL, CLOCK, PERs (including PER1, PER2, PER3) and CRYs (including CRY1, CRY2, CRY3). The clock control genes, including RORs (including RORA, RORB, RORC) and NR1Ds (including NR1D1, NR1D2), modulate the expression of core clock genes. The CLOCK‐ARNTL complex controls transcription of PERs and CRYs while accumulated PERs and CRYs in turn repress CLOCK‐ARNTL complex activity with the assistance of TIMELESS. This negative feedback contributes to the formation of circadian rhythm. We collected circadian clock genes (CCGs), including core clock genes and clock control genes identified in previous studies for further analysis.^6^, ^8^, ^9^

Disrupted circadian rhythms are common in several cancers including lung cancer, breast cancer and colorectal cancer,^10^, ^11^, ^12^, ^13^ where they influence tumour angiogenesis, apoptosis and proliferation.^14^, ^15^, ^16^ For instance, ARNTL induces cell cycle arrest in tumour cells by affecting p53 function^17^ and represses tumour cell invasion through PI3K signalling pathway.^18^ CLOCK promotes high‐grade glioma proliferation and migration ^19^ and modulates the cellular response to DNA damage in colorectal cancer.^20^ Nevertheless, the role of CCGs in glioma is still largely unknown.

In this study, we proved the prognostic model based on CCGs can be applied to predict patient survival in the TCGA dataset (Figure S1A). The results were also validated in four independent datasets, including three CGGA datasets and one GSE108474 dataset. Two prognostic model we built both indicated CCG expression can predict patients’ survival outcome. We scored samples with the LASSO regression analysis, and high scoring tumours manifested worse survival outcome. The scoring system can also be applied to other tumours indicating the importance of CCGs in tumour progression. To qualified the role of our scoring system, a nomogram was established for clinical application.

As for how CCG expression affects glioma progression, higher scoring tumour usually along with higher immune infiltration ratio and more sensitivity to immunotherapy. Notably, CGG expression can affect tumour progression by inhibiting tumour cell proliferation. TIMELESS is a critical gene in regulating the expression of core CCGs. By silencing TIMELESS not only dysregulated CCG expression but also arrested cell cycle at the G0/G1 phase. Therefore, abnormal CCGs expression suppressed tumour cell proliferation according to CCK8 assay and colony‐forming assay. In general, abnormal CCG expression affects tumour prognosis through influencing tumour immune landscape and tumour cell proliferation.

## 2 MATERIALS AND METHODS

### 2.1 Data processing

RNA‐seq data and corresponding clinical information were obtained from the TCGA (https://cancergenome.nih.gov/), CGGA (referred to as mRNAseq_325 (CGGA1) dataset, mRNA‐array (CGGA2) dataset and mRNAseq_693 (CGGA3); http://www.cgga.org.cn/) and NCBI GEO (GSE108474; https://www.ncbi.nlm.nih.gov/gds) (Table S1). TCGA data were used as a training set and others were used for validation. Among these, TCGA, CGGA1 and CGGA3 are RNA sequencing data and CGGA2 and GSE108474 are RNA microarray data. Heatmaps were used to illustrate the expression profile of CCGs.

Single‐cell RNA‐Seq data from GSE139448 were downloaded and processed using R package ‘Seurat’.^21^ ‘NormalizeData’ and ‘FindVariableGenes’ were conducted to normalized data and identified 2000 highly variable genes, respectively. Three glioma samples (GBM27, GBM28 and GBM29) from GSE139448 were merged for further analysis. Perform principal component analysis was conducted using the ‘FindNeighbors’ and ‘FindClusters’ function. Expression profiling of the genes composing the riskScore were depicted by violin plot using the function ‘vlnplot’. Differentially expressed genes were filtered between high‐ and low‐risk groups and defined as significant when |logFC| > 1 and *P*‐value < .01. Pseudo‐cell analysis was introduced to increase the correlation between gene number and gene expression.^22^, ^23^

### 2.2 Least absolute shrinkage and selection operator (LASSO) Cox regression

Genes filtered by univariate Cox analysis were further applied to the LASSO Cox regression and their coefficients were calculated in accordance with the highest lambda value.^24^ The scoring system was established on the coefficients of genes, the result of which we termed the ‘riskScore’. Patients were characterized into high‐ and low‐risk groups according to the median value of the riskScore. The formula for riskScore calculation is as follows:

$$\begin{array}{c} \text{riskScore}=0.30145833*\text{ARNTL}+0.08807400*\text{ARNTL2}+0.14204612*\text{BHLHE40}+\\ \;\;\;\left(‐0.36049846*\text{CRY2}\right)+\left(‐0.06305725*\text{CSNK1E}\right)+\left(‐0.03387665*\text{HLF}\right)+\\ \;\;\;\left(‐0.04254696*\text{NR1D2}\right)+\left(‐0.08047881*\text{PER3}\right)+0.01191781*\text{RORC}+\\ \;\;\;0.17275780*\text{TIMELESS} \end{array}$$

### 2.3 Consensus clustering analysis

Samples were divided into different groups by consensus Clustering analysis with the R package ‘Consensus Cluster Plus’ to create the Cluster model.^25^, ^26^ The optimum amount of Clusters was decided in line with the cumulative distribution function plots and consensus matrices.^27^

### 2.4 Biological function prediction

Gene Ontology (GO) analysis and Kyoto Encyclopedia of Genes and Genomes (KEGG) analysis on high‐ or low‐risk group were conducted by GSVA analysis, and relevant information was download from Molecular Signature Database (MSigDB).^28^, ^29^ Results from the GSVA analysis with a false discovery rate < 0.05 were considered significant. Additionally, relationships between high‐ or low‐risk group and multiple gene sets from the MSigDB were calculated using GSEA analysis.

Genes related to T cell–mediated immunity (http://www.gsea‐msigdb.org/gsea/msigdb/cards/GO_REGULATION_OF_T_CELL_MEDIATEDIMMUNITY) and cell cycle DNA replication (http://www.gsea‐msigdb.org/gsea/msigdb/cards/GO_CELL_CYCLE_DNAREPLICATION) were selected from MSigDB. The immune infiltration landscape was investigated by the ssGSEA algorithm as previous depicted.^30^, ^31^ The ESTIMATE algorithm was used to assess the composition of tumour microenvironment.^32^

### 2.5 Treatment prediction

Candidate drugs target to glioma were selected by using the CMap (https://portals.broadinstitute.org/cmap/).^33^ DEGs between high‐ and low‐risk group were identified by the limma package with setting |logFC| >1 and *P*‐value < .05. Then, correlation between DEGs and riskScore was illustrated by Spearman's correlation, and DEGs with correlation coefficient > 0.5 were filtered for analysis in CMap. The enrichment score of candidate drugs was calculated by CMap. Positive score suggests drug promotes the input signature while negative score inhibits. Therefore, we selected drugs with negative score <−0.9 and *P*‐value < .05 as candidate.

### 2.6 Genetic variation analysis in glioma

Genetic variation information in glioma, including single nucleotide polymorphisms (SNPs) and copy number variations (CNVs), was downloaded from the TCGA database. GISTIC (version 2.0) was used to explore copy number information of alteration peaks in the high‐ or low‐risk groups.^34^

### 2.7 Survival analysis and nomogram

The Kaplan‐Meier analysis was applied to generate survival curves, and the validity was assessed by the log‐rank test. Receiver operating characteristic (ROC) curve and the area under the curve (AUC) were introduced to contrast the predictive ability of different model.

Univariate and multivariate Cox regression analysis was used to filter prognostic variables (*P*‐value < .05). Consequently, these variables were verified by the Schoenfeld test to construct a nomogram with the r package ‘RMS’.^35^, ^36^ The calibration curve and ROC were used to evaluate the accuracy of the nomogram for OS prediction.

### 2.8 Cell culture

Glioma cells, U251 and T98G, were acquired from the Chinese Academy of Sciences. Cells were cultured in the DMEM at 37℃ with 5% CO_2_. Cells were divided into control group, siRNA‐negative control group and siRNA (siRNA‐871, siRNA‐1032, siRNA‐2526) group.

### 2.9 Western blot assay

Proteins were extracted using RIPA, and the concentration of proteins was determined by BCA protocol. Antibody of TIMELESS (Proteintech Cat# 14421‐1‐AP, RRID: AB_2201962) and β‐actin (Proteintech Cat# 66009‐1‐Ig, RRID: AB_2687938) was added for Western blot assay. HRP secondary antibody (Proteintech Cat# SA00001‐1, RRID: AB_2722565; Proteintech Cat# SA00001‐2, RRID: AB_2722564) was added at the second day. The expression of TIMELESS in the siRNA‐1032 group was not significantly silenced; thereby, siRNA‐1032 was not used in the following experiment.

### 2.10 RT‐qPCR

RNA extraction was performed followed the Trizol protocol. Extracted RNA integrity was verified before PCR. Former primer and reverse primer of CCGs, designed by primer 5, were added to a PCR mix. PCR initiated with 95℃ for 10 minutes, followed with 40 cycles (each cycle consists of 95℃ for 15 seconds and 60℃ for 30 seconds).

### 2.11 Cell proliferation and cell cycle

Colony‐forming assay was performed to show the relationship between TIMELESS and cell viability. Cells were plated into 6‐well plates and fixed after culturing for two weeks. The OD value at 550 nm was measured after staining and decolouring cells.

To detecting cell cycle alternation, single‐cell suspension (1*10^6^ cells per millilitre) was fixed and harvested on the second day. Then, cells were loaded on flow cytometer and the proportion of cells with different cell cycle was calculated using ModFit LT (version 5.0).

For CCK8 assay, cells (1*10^3^) were seeded on 96‐well plates, and the OD value at 450 nm was measured at different time.

### 2.12 Drug sensitivity

Cells were also divided into different groups with different drug concentration (10, 20 and 30 μmol/L). Agonists of NR1D1 and NR1D2, SR9009 (Cat# HY‐16988) and SR9011 (Cat# HY‐16989), were purchased from MedChemExpress (Shanghai, China).^37^ Cells (2*10^3^ per well) were seeded on the 96‐well plate, and agonists were added with 10, 20 and 30 μmol/L at final concentration. Cell sensitivity to drugs was also evaluated by the CCK8 assay.

### 2.13 Statistical analysis

Statistical analysis was carried out using r (version 3.6.2) and graphpad prism (version 8.0). Wilcoxon rank‐sum test was used to compare two groups. One‐way ANOVA was used to compare multiple groups. Pearson's correlation analysis was conducted to calculate correlation coefficient. NS: not statistically significant; *P < .05; **P < .01; ***P < .001. And *P*‐value < .05 was considered statistically significant.

## 3 RESULTS

### 3.1 Expression profile of circadian clock genes and clinical features of glioma

**FIGURE 1:**
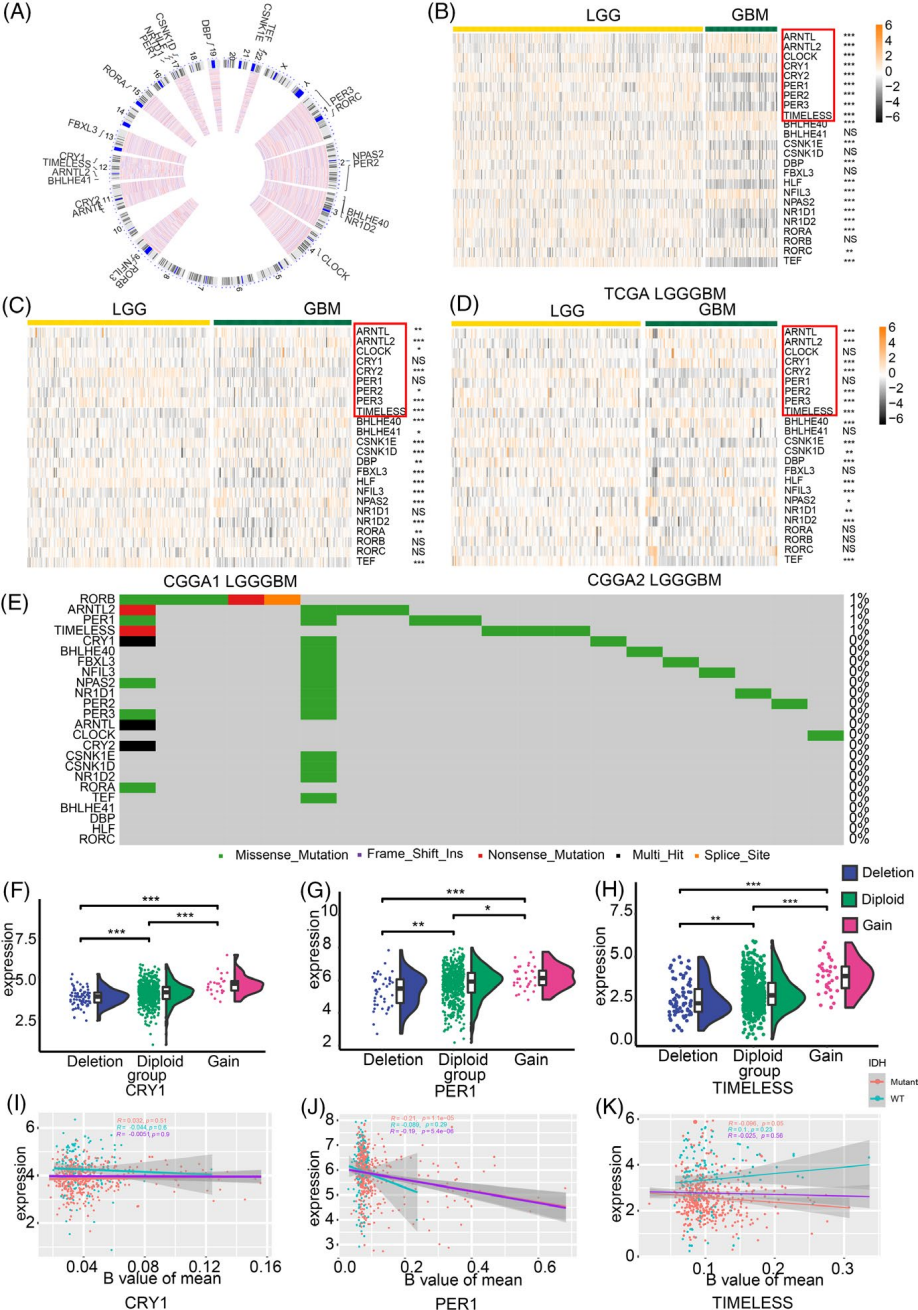
Landscape of CCG expression profile. (A) The chromosomal locations of CCGs. Heatmap illustrating CGG expression differences between LGG and GBM in the TCGA (B), CGGA1 (C) and CGGA2 (D) datasets. (E) SNPs present in CGGs of all samples from the TCGA dataset. CNVs for, CRY1 (F), PER1 (G) and TIMELESS (H). The DNA methylation status of CRY1 (I), PER1 (J) and TIMELESS (K) along with the status of IDH. The core clock genes are labelled with red. NS: not statistically significant; *P < .05; **P < .01; ***P < .001

We selected 24 CCGs that contribute to circadian rhythm and summarized their location on chromosomal^38^, ^39^, ^40^ (Figure 1A). A protein‐protein interaction network of these 24 genes indicates the complicated regulation of circadian rhythm. The core clock genes TIMELESS, CLOCK, ARNTL, PERs and CRYs are located at the hub of the network (Figure S1B). Expression profile of CCGs between normal brain tissue and gliomas was introduced the heatmap. Expression difference was noticed such as ANRTLs, PERs, CLOCK, TIMELESS, BHLHEs implying disordered circadian rhythm in gliomas (Figure S1C).

Then, the expression profile of CCGs in glioma was also depicted. Higher expression of ARNTL, ARNTL2, CRY1, NFIL3, NPAS2 and TIMELESS and lower expression of CLOCK, CRY2, PER1, PER2, PER3, CSNK1E, HLF, NR1D1 and TEF were observed in GBM compared to LGG tissues from TCGA (Figure 1B). However, expression of some core clock genes like CLOCK, PER1 and CRY2 showed no significant difference in the CGGA1 (Figure 1C) or CGGA2 dataset (Figure 1D). Most of CCGs did not show significant expression difference between Grade II and Grade III glioma such as BHLHEs in the TCGA (Figure S2A) and CGGA1 (Figure S2B) dataset or ARNTLs, CRYs in the CGGA2 dataset (Figure S2C). IDH status is another critical clinical feature to predict patient's survival outcome. As illustrated, less no significant expression difference genes were noticed than the comparison of grade II and grade III glioma. (Figure S2D‐F). Therefore, disordered circadian rhythm may be highly associated with glioma prognosis and tumour progression.

SNPs and CNVs about CCGs were also explored. As illustrated, samples carried with mutated RORB, ARNTL, PER1 and TIMELESS only on account of 1% of all samples from the TCGA dataset (Figure 1E). Meanwhile, CNV profile of core CCGs was able to explain the abnormal expression of CCGs (Figures 1F‐H and S3A‐F). For example, the expression of ARNTL, ARNTL2, TIMELESS, CRY1 was increased in glioma relative to normal brain tissue, which is corresponding with the CNV profile of CCGs. However, PER2 expression and PER3 expression were lower in glioma than normal brain tissue, which is conflict with the CNV profile. Then, DNA methylation of CCGs was analysed in all samples, IDH wild‐type glioma, and IDH mutate glioma (Figures 1I‐K and S3G‐L). Negative correlation between the methylation status of core CCGs and gene expression was noticed. Besides, the methylation ratio of core CCGs in all samples was positively correlated with IDH wild‐type group and IDH mutate group. In summary, the analysis based on SNPs, CNVs, and DNA methylation indicated that abnormal expressed CCGs existed in glioma implying this expression alternation related to glioma progression.

### 3.2 The Cluster model

Clustering consensus analysis was used to construct the Cluster model based on CCGs. In the TCGA database (Figure S4A), samples were divided into two clusters (Cluster1 and Cluster2), and the same strategy was applied to the CGGA1 (Figure S4B), CGGA2 (Figure S4C), CGGA3 (Figure S4D) and GSE104878 (Figure S4E) datasets. The number of Clusters (k = 2) was determined by cumulative distribution function curves and consensus matrixes.

**FIGURE 2:**
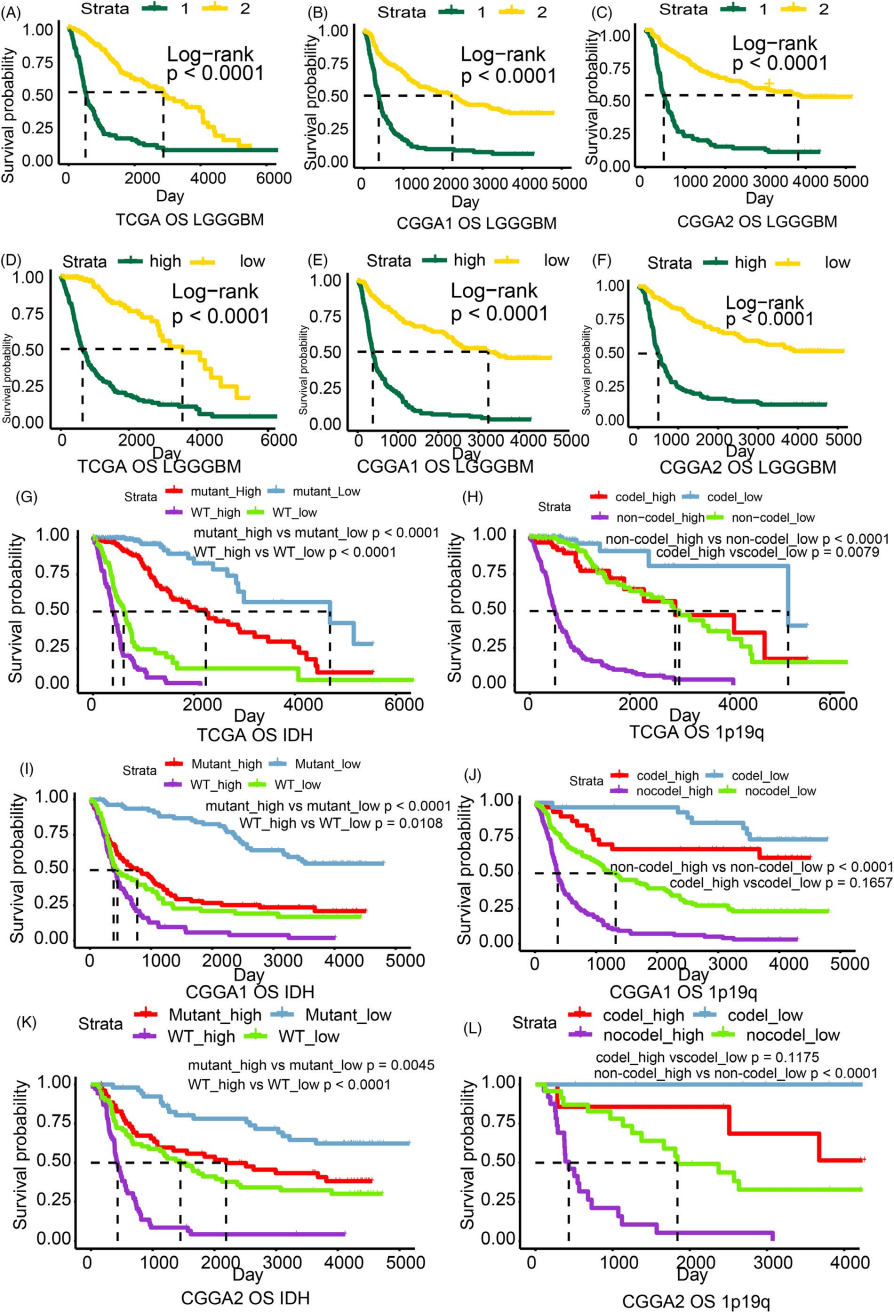
Survival analysis based on the cluster model and riskScore model. Survival analysis of the LGGGBM cohort based on the cluster model in the TCGA (A, *P*‐value < .0001), CGGA1 (B *P*‐value < .0001), CGGA2 (C, *P*‐value < .0001) datasets. Survival outcomes for high‐ and low‐risk groups in the LGGGBM cohort from the TCGA (D, *P*‐value < .0001), CGGA1 (E, *P*‐value < .0001) and CGGA2 (F, *P*‐value < .0001) datasets. Survival analysis for high‐ and low‐risk groups based on subgroups characterized by IDH status and 1p19q status from the TCGA (G: mutant_high vs mutant_low < 0.0001, WT_high vs WT_low < 0.0001; H: noncodel_high vs noncodel_low < 0.0001, codel_high vs codel_low = 0.0079), CGGA1 (I: mutant_high vs mutant_low < 0.0001, WT_high vs WT_low = 0.0108; J: noncodel_high vs noncodel_low < 0.0001, codel_high vs codel_low = 0.1657) and CGGA2 (K: mutant_high vs mutant_low = 0.0045, WT_high vs WT_low < 0.0001; L: noncodel_high vs noncodel_low < 0.0001, codel_high vs codel_low = 0.1175) datasets

Additionally, overall survival analysis was conducted to compare the prognostic characteristics between the clusters. Cluster2 manifested longer overall survival relative to Cluster1 in the LGGGBM cohort in TCGA (Figure 2A), CGGA1 (Figure 2B) and CGGA2 (Figure 2C) datasets. However, opposing results were obtained from the CGGA3 and GSE108474 datasets (Figure S5A,B). Survival analysis was also performed on the LGG and GBM cohorts separately. Results from the LGG cohort were similar to the LGGGBM cohort, but no significant difference was observed in the GBM cohort (Figure S5C‐G).

### 3.3 Establishment of a prognostic riskScore model derived from CCG expression

Nineteen of twenty‐four CCGs were found to be related to glioma prognosis (*P*‐value < .05) by univariate Cox regression analysis (Table S2). Application of the more stringent least absolute shrinkage and selection operator (LASSO) Cox regression identified 10 prognosis‐related genes. We used the coefficients from the LASSO model to calculate a prognostic metric which we termed the ‘riskScore’ (Figure S6A‐C). Samples were then classified into high‐ and low‐risk groups according to the median value of the riskScore.

Patients with a high riskScore showed statistically shorter survival time than those with low riskScores in the TCGA LGGGBM cohort (Figure 2D). Similar conclusions can be drawn from the validation cohort (Figures 2E,F and S6D,E). In the LGG cohort, high riskScore also indicated worse survival, while no significant overall survival difference was observed in the GBM cohort (Figure S6F‐J). Subgroup survival analysis based on IDH status, 1p19q status, MGMT status and radiotherapy was also conducted, and patients with a higher riskScore exhibited worse survival outcomes in the TCGA and validation datasets (Figures 2G,L, S6K,L, and S7). Together, these findings clearly demonstrate the prognostic value of the CCG‐derived riskScore in multiple glioma datasets and among clinically relevant patient subgroups.

Then, the distribution of riskScore was further analysed. High riskScore samples were high‐grade glioma, IDH wild‐type glioma, 1p19q non‐codel glioma, MGMT unmethylated glioma, aggressive glioma (classical and mesenchymal) and worse treatment outcome glioma in the training and validation cohort (Figures S8 and S9).

### 3.4 Single‐cell transcriptomic context of CCGs and the riskScore

**FIGURE 3:**
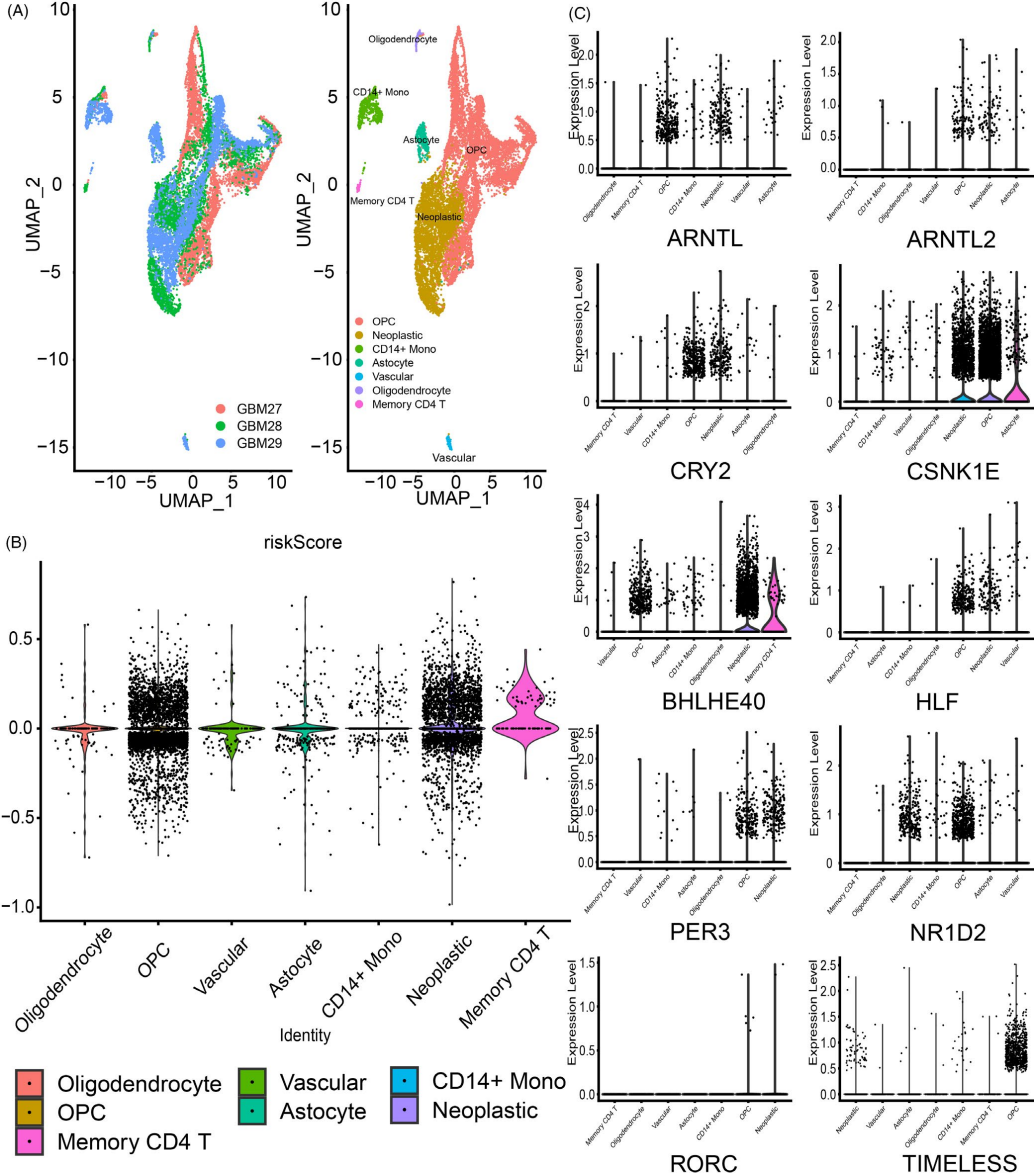
CCG expression and riskScore profile based on single‐cell sequencing analysis. (A) Composition and distribution of single cells from GSE139448. (B) RiskScore was calculated for each cell and their distribution was illustrated. Each cell type is ranked by their average riskScore. (C) The expression profile of riskScore‐related genes for each cell is also presented. Each cell type is ranked by their average expression value

To further verify the relationship between CCGs and riskScore in glioma, single‐cell RNA sequencing data from GBM was employed (Figure 3A). We calculated riskScore for each cell, and they were ranked by their average riskScore (Figure 3B). Notably, memory CD4 T cells and tumour cells showed higher riskScore than other cell types. The distribution of riskScore related to CCGs was visualized (Figure 3C). RiskScore of tumour cells and stromal cells like oligodendrocyte precursors and astrocyte was higher than other cell types. Together, those results proved that disordered circadian rhythm widely existed in tumour cells and cells in tumour microenvironment. Along with previous results, further supported disordered circadian rhythm influenced glioma progression.

### 3.5 Epigenetic alternation in patients within the high or low riskScore groups

**FIGURE 4:**
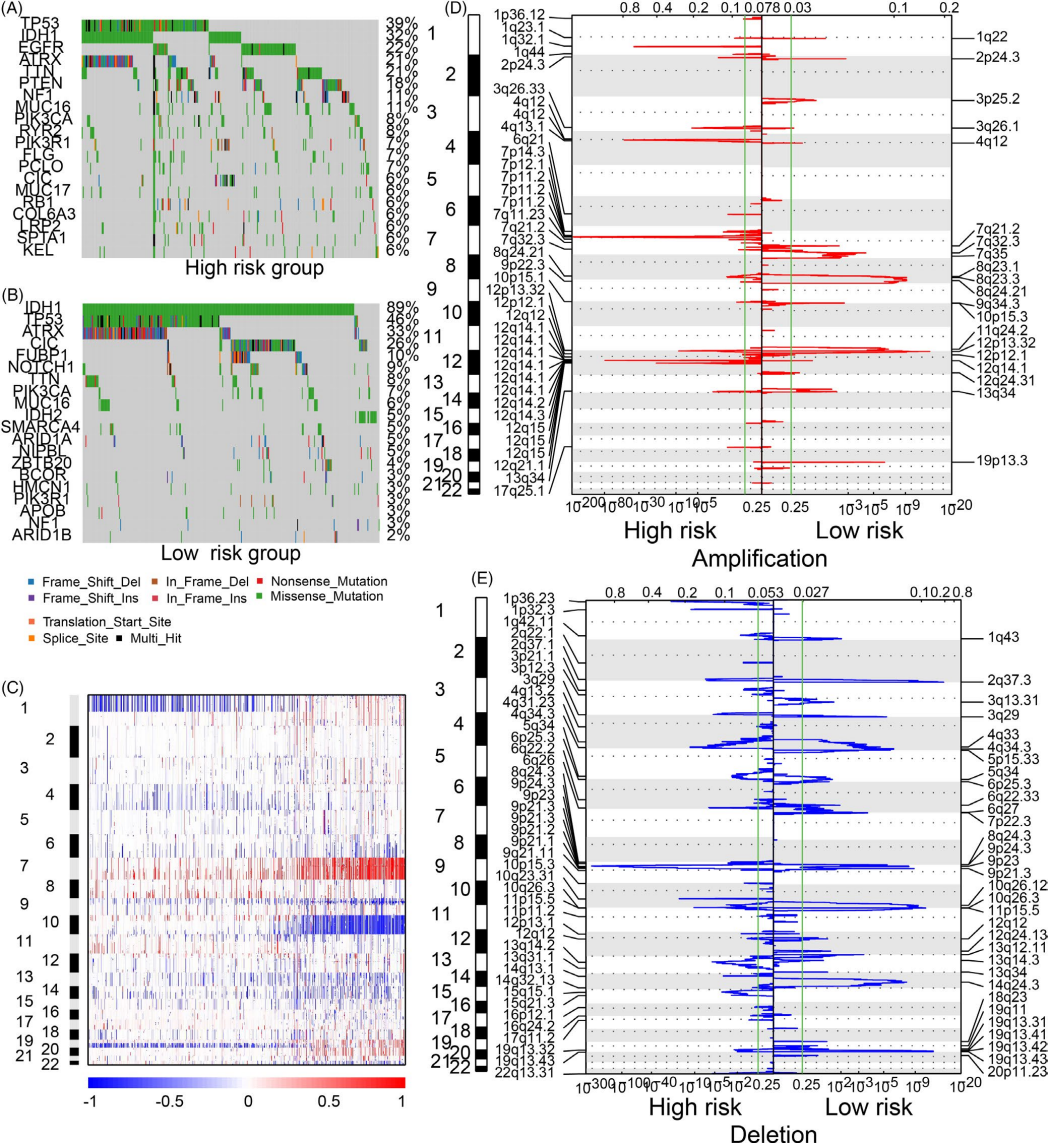
CNV and SNP difference between high‐ and low‐risk group. Landscape of SNPs for high (A)‐ and low‐risk groups (B). Overall CNVs (C) and CNVs in high (D)‐ or low‐risk groups (E) stratified by riskScore

SNPs and CNVs in high‐ and low‐risk groups were investigated, respectively. In general, incidence of SNPs was more common in the low‐risk group relative to high‐risk group (Figure 4A,B). A higher degree of TP53 (46% vs 39%), IDH1 (89% vs 32%) and ATRX (33% vs 21%) mutations was observed in low‐risk compared to high‐risk groups. In the high‐risk group, EGFR, PTEN, NF1 were most common mutations.

For CNVs, chromosome 1 deletions were more likely to be enriched in samples with lower riskScore while the ratio of chromosome 7 amplification and chromosome 10 deletion were paralleled by an increased riskScore (Figure 4C). Amplified regions including 7p11.2 (EGFR) and 12q14.1 (CDK4) were enriched in high‐risk groups, while 11q24.2 (PARP11) and 12p13.32 (hsa‐mir‐3167) were observed in the low‐risk group. Meanwhile, deletion of regions including 9p21.3 (CDKN2A) and 1p36.23 (ERRFI1) was detected in the high‐risk group (Figure 4D), and 2q37.3 (hsa‐mir‐3133) and 11p15.5 (hsa‐mir‐4298) were common deletion regions in the low‐risk group (Figure 4E).

### 3.6 A riskScore‐derived nomogram efficiently predicts glioma outcomes

**FIGURE 5:**
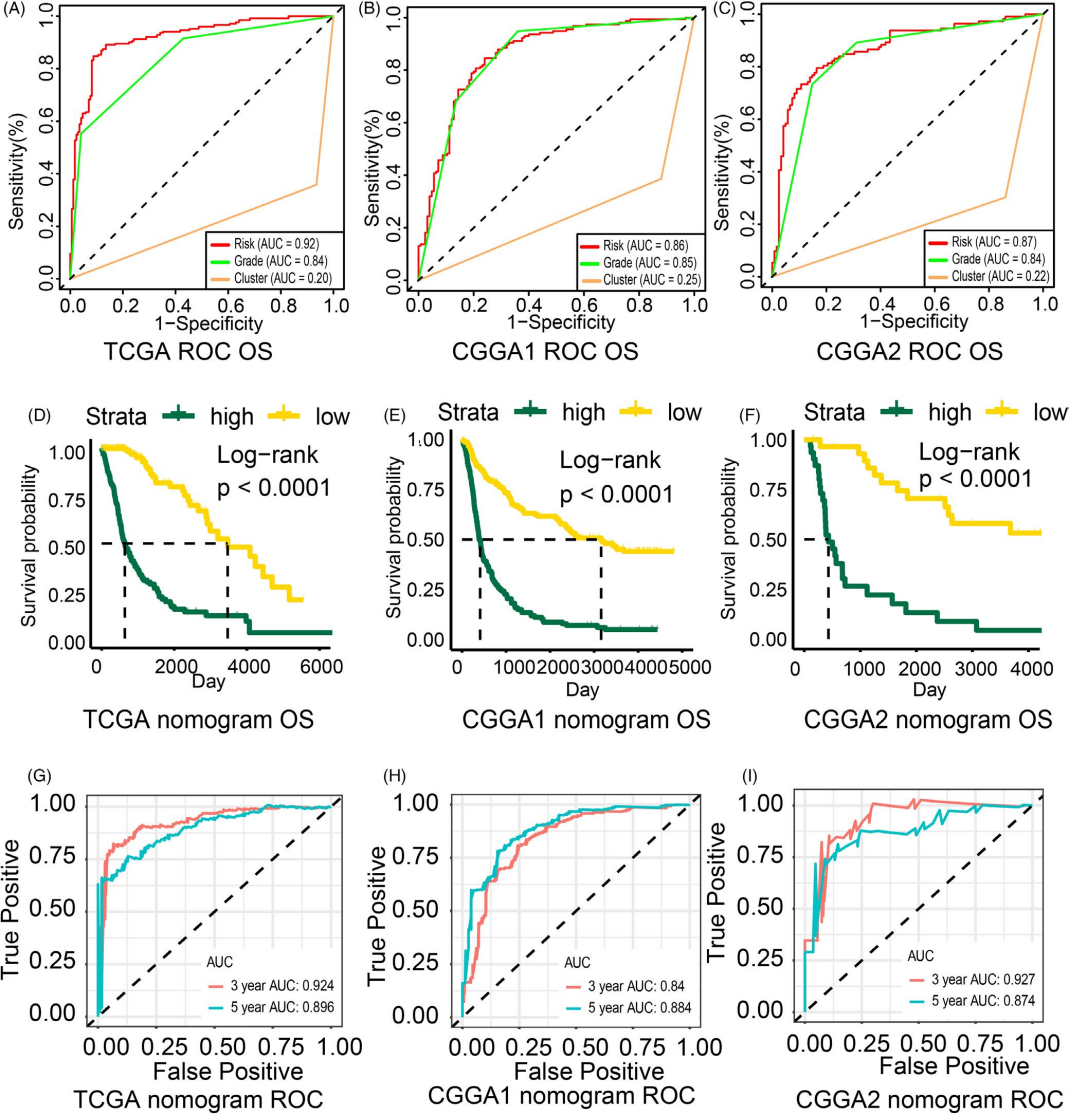
Nomogram based on riskScore and clinical features. ROC curve generated to predict sensitivity and specificity of the riskScore for overall survival based on the TCGA (A), CGGA1 (B) and CGGA2 (C) datasets. Survival analysis based on high or low points group according to nomogram based on the TCGA D, *P*‐value < .0001), CGGA1 (E, *P*‐value < .0001) and CGGA2 (F, *P*‐value < .0001) datasets. ROC curve for 3‐year and 5‐year overall survival based on applying the nomogram to the TCGA (G, AUC: 3‐year: 0.924, 5‐year: 0.896), CGGA1 (H, AUC: 3‐year: 0.84, 5‐year: 0.884) and CGGA2 (I, AUC: 3‐year: 0.927, 5‐year: 0.874) datasets

Receiver operating characteristic curves (ROC curve) were constructed to compare the prognostic ability of the Cluster model, riskScore model and tumour pathological grade. Among these metrics, the riskScore was the best predictor of OS (Figure 5A,C) while Cluster was the worst. Next, we selected riskScore along with relevant clinical features, including age, tumour grade, cancer, 1p19q, IDH, MGMT, glioma subtype (mesenchymal, classical, proneural, neural)^41^ to conduct univariate and multivariate Cox regression analysis (Table S3). Factors with *P*‐value < .001 were considered statistically significant. Based on the findings of this analysis, riskScore (HR = 2.331), 1p19q with non‐codel status (HR = 1.810), and age (HR = 1.040) were integrated to construct the nomogram (Figure S10A).

Calibration curves were used to validate the accuracy of the nomogram fabricated from the TCGA and CGGA1 dataset (Figure S10B‐D). Dividing patients into high or low points groups based on the nomogram indicated that high point samples from TCGA, CGGA1 and CGGA2 databases have worse survival outcomes (Figure 5D‐F). Corresponding ROC and AUC were also calculated (Figure 5G‐I). Notably, the AUC index for 3‐year and 5‐year overall survival was 0.924 and 0.896, respectively, in the TCGA dataset. Moreover, the AUC index for 3‐year OS and 5‐year overall survival in other datasets was also more than 0.80 indicating the high accuracy of this nomogram. Together these findings highlight the powerful prognostic ability of the riskScore‐derived nomogram model in multiple glioma datasets.

### 3.7 Association between tumour progression pathways and riskScore

**FIGURE 6:**
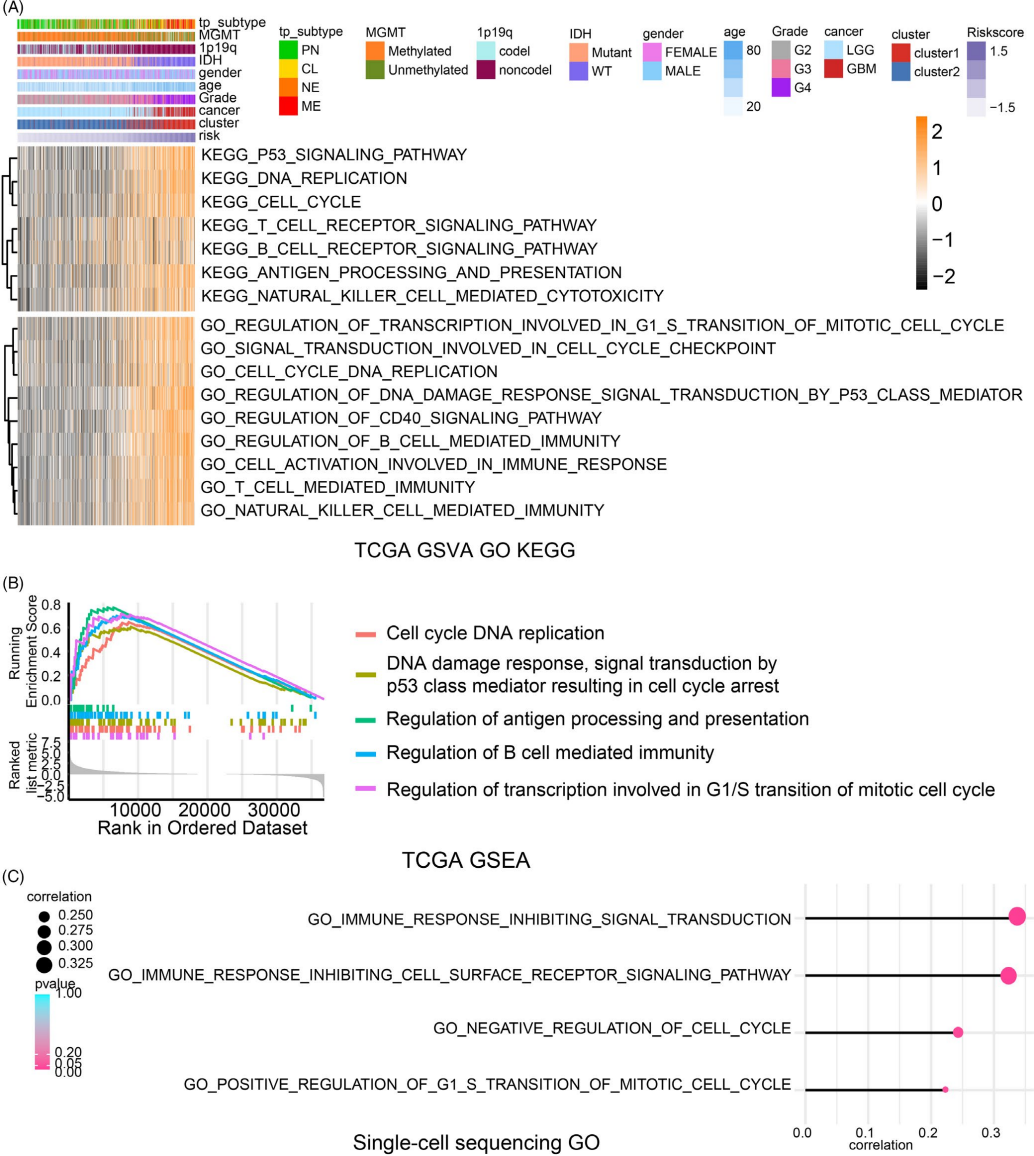
Biofunction prediction according to riskScore. (A) Heatmap of GO and KEGG analysis based on riskScore in the TCGA dataset. (B) GSEA analysis in the TCGA dataset according to riskScore. (C) GO analysis based on single‐sequencing analysis

In the TCGA dataset, GO and KEGG enrichment analysis based on GSVA analysis (Figures 6A, and S11A) and GSEA analysis (Figure 6B) suggested that CCGs modulate cell cycle and immune infiltration. Besides, GO analysis based on GSVA analysis (Figure 6C) and based on differential expression genes between high and low riskScore group (Figure S11B) in the single‐cell sequencing analysis also supported that conclusion.

**FIGURE 7:**
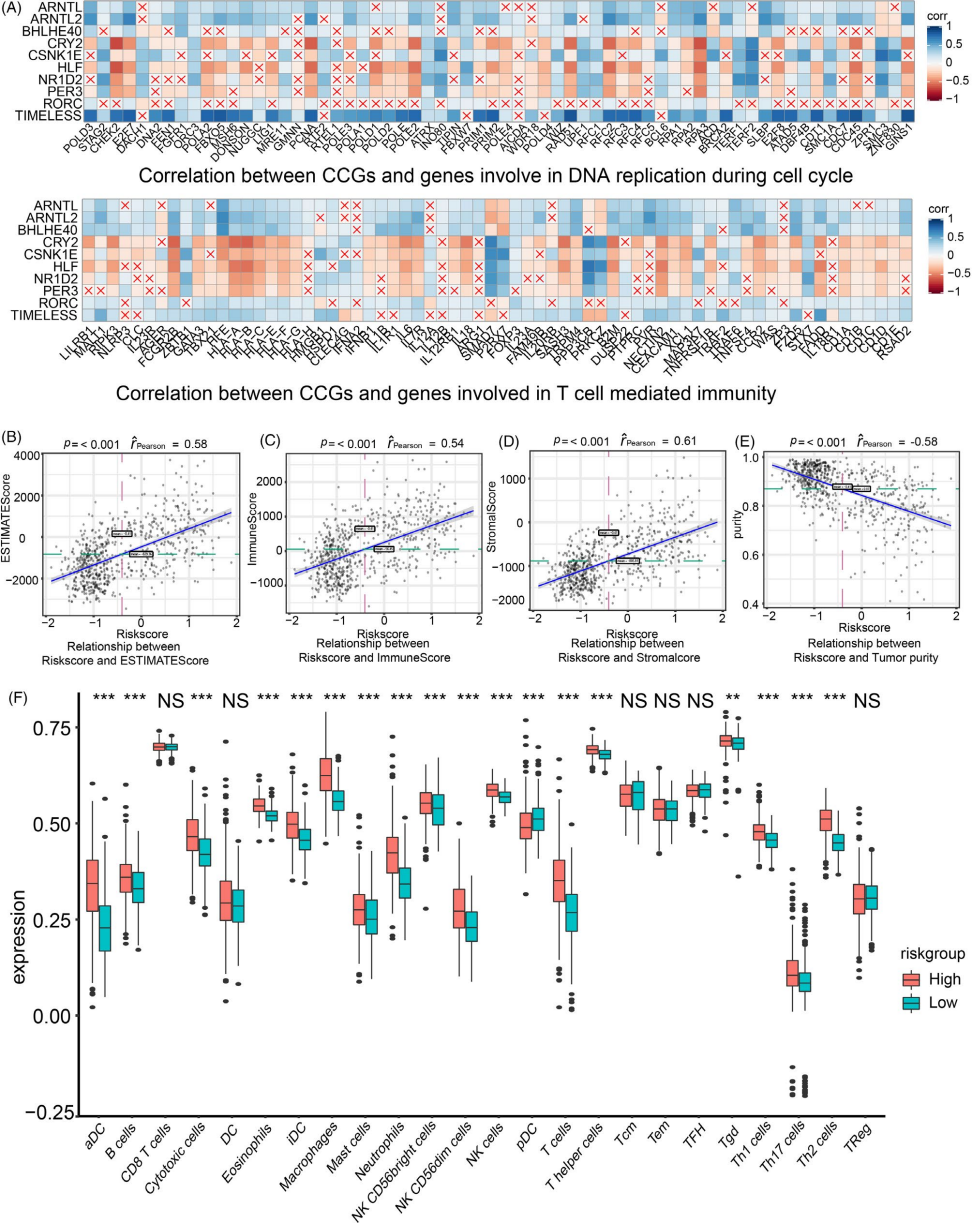
Immune landscape based on the riskScore model and prediction of tumour response to immunotherapy. (A) Correlation between RiskScore‐related genes and genes involved in T cell–mediated immunity and DNA replication during cell cycle, respectively. ESTIMATE score (B, *r* = 0.58, *P*‐value < .001), immune score (C, *r* = 0.54, *P*‐value < .001), stromal score (D, *r* = 0.61, *P*‐value < .001), tumour purity (E, *r* = −0.58, *P*‐value < .001) and their correlation with riskScore. (F) Expression profile of different types of infiltrating immunocytes based on riskScore. Upregulated (red stripe) and downregulated (blue stripe) immune cells are presented according to immunocyte infiltration analysis. NS: not statistically significant; *P < .05; **P < .01; ***P < .001

Therefore, we investigated the correlation between riskScore‐related CCGs and genes involved in T cell–mediated immunity and DNA replication during cell cycle (Figure 7A). We found that CRY2, CSNK1E, HLF, NR1D2 and PER3 were negatively correlated to T cell–mediated immunity while others were positively correlated. Additionally, CRY2, HLF, NR1D2 and PER3 were negatively correlated with genes controlling cell cycle DNA replication while others were positive. Similar correlation with T cell–mediated immunity (Figure S12A) and cell cycle DNA replication (Figure S12B) was also verified in CGGA1 and CGGA2 datasets.

We also predicted drug sensitivity according to riskScore. DEGs (n = 545, 90 upregulated, 455 downregulated) between high‐ and low‐risk group were identified and analysed by CMap to filter potential drug targets. Podophyllotoxin and nystatin manifested negative correlation with riskScore indicating their role as potential drugs target to glioma (Table S4).

### 3.8 Immune infiltration profile based on riskScore

We next assessed the association between riskScore and immune infiltration in the TCGA dataset. The ESTIMATE algorithm was first introduced, and results suggested that higher riskScore was associated with a higher immune score and stromal score and lower tumour purity (Figure 7B‐E). These findings were validated in the other datasets (Figure S13A,B) implying the immune landscape difference between high and low riskScore samples.

Anti‐tumour immunocytes including T cells, cytotoxic T cells, NK cells, macrophages and immunosuppressive cells including Th2 cells, apoptotic dendritic cells, immature dendritic cells were enriched in high riskScore group (Figures 7F and S13C,D). The distribution (Figure S14A) and correlation (Figure S14B) of riskScore and immunocytes infiltration were also analysed. Together, more immunocytes were infiltrated in high riskScore group.

### 3.9 CCGs affect tumour cell proliferation

**FIGURE 8:**
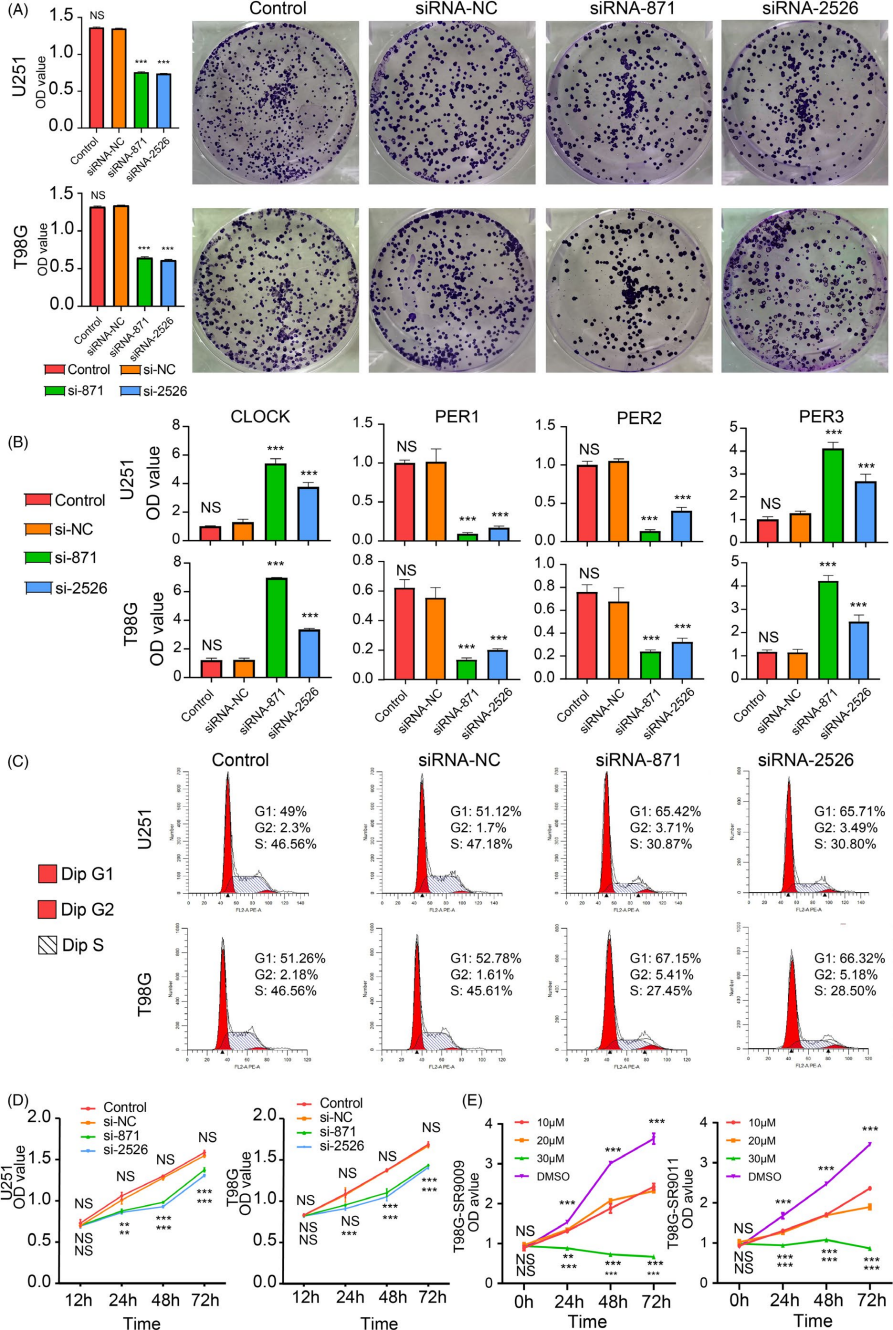
CCGs affect glioma cells proliferation. (A) The colony‐forming assay of U251 and T98G supported the ability of cell viability is worse in the TIMELESS‐siRNA group. (B) qRT‐PCR suggests the expression of CLOCK; PER3 is increased after silencing TIMELESS expression. In the meantime, PER1 and PER2 are also downregulated in the TIMELESS‐siRNA group. (C) Flow cytometry indicated cell cycle was arrested at the G0/G1 phase. (D) CCK8 assay suggests the ability of cell proliferation is affected in the siRNA group than the control group. (E) T98G showed sensitivity to SR9009 and SR9011 by comparing to the DMSO group. Data were presented as mean + SD

Based on the result from the LASSO regression analysis and previous researches, we selected TIMELESS for further analysis. We first verified the expression of TIMELESS protein by performing the Western blot assay, and siRNA‐1032 was discarded in the following experiment due to its low efficiency (Figure S14C). The colony‐forming assay suggested that the viability of tumour cells in the TIMELESS‐siRNA group was inhibited (Figure 8A). By silencing TIMELESS expression can alter the expression of CLOCK and PERs implying circadian rhythm was interrupted (Figure 8B).

Then, the flow cytometry supported cell cycle was arrested at the G0 phase in the TIMELESS‐siRNA group compare to the negative control group (Figure 8C). In the CCK8 assay, glioma cell proliferation was significantly suppressed in the TIMELESS‐siRNA group relative to the negative control group (Figure 8D). Besides, agonist of NR1D1 and NR1D2 was proved to influence circadian rhythm in previous researches.^42^, ^43^ In this work, T98G showed sensitivity to SR9009 and SR9011 implying interrupted circadian rhythm affected cells proliferation; in the meantime, U251 proliferation was also inhibited by SR9009 and SR9011 (Figure 8E and S14D). Therefore, cell proliferation was affected by interfering circadian rhythm.

## 4 DISCUSSION

Circadian rhythm affects physiological activities of human body. However, it was interrupted in tumour such as breast cancer and colorectal cancer.^10^, ^11^, ^12^, ^13^ Dysregulated CCG expression was associated with glioma progression^19^, ^44^, ^45^ and glioma stem cells.^46^ In this work, we constructed prognostic model and proved disordered circadian rhythm is associated with glioma immune landscape and cell proliferation.

Two prognostic model, the cluster model and the riskScore model, both confirmed CCG expression is associated with glioma prognosis in this work. The riskScore model is confirmed as the best method to predict glioma prognosis. A high accuracy clinical prognostic model, nomogram, was constructed based on riskScore, age and 1p19q status. Besides, the riskScore model can also be applied to other tumours in the TCGA dataset, including adrenocortical carcinoma, oesophageal carcinoma, kidney chromophobe, kidney renal clear cell carcinoma, liver hepatocellular carcinoma, lung adenocarcinoma, lung squamous cell carcinoma, ovarian serous cystadenocarcinoma, pancreatic adenocarcinoma, rectum adenocarcinoma, uterine corpus endometrial carcinoma and uveal melanoma (Figure S15).

We also predicted podophyllotoxin and nystatin may be sensitivity drug for high riskScore glioma patients. Notably, previous study supported nystatin was associated with the ratio of immunosuppressive myeloid cells^47^ and deoxypodophyllotoxin can inhibit glioma progression.^48^, ^49^ Therefore, the riskScore model can also be applied to predict potential drugs. Together, we constructed high accuracy, high confidence and widely application model for evaluating the association between circadian rhythm and tumour.

The maintenance of circadian rhythm depends on the interaction between TIMELESS and PERs and interrupting the expression of them cause disordered circadian.^50^, ^51^ In the TIMELESS‐siRNA group, CLOCK and PER3 expression increased while PER1 and PER2 decreased implying interfered circadian rhythm. In the meantime, cell cycle was blocked at G0 phase and cell proliferation was inhibited. Therefore, TIMELESS can promote glioma progression through affecting cell cycle and cells proliferation. Previous studies also mentioned that TIMELESS can affect tumour progression by influencing cells cycle including liver cancer, cervical cancer, nasopharyngeal carcinoma, breast cancer.^52^, ^53^, ^54^, ^55^ Besides, PERs and CLOCK also promote cells proliferation by modulating cell cycle.^56^, ^57^, ^58^, ^59^ Furthermore, agonist of NR1D1 and NR1D2 has been proved can be interrupted circadian rhythm and inhibited glioma proliferation.^42^, ^43^ In our work, similar conclusion was also obtained. Together, disordered CCGs can promote tumour progression by interfering normal cell cycle.

The composition of immunocytes in tumour microenvironment is also known to affect glioma prognosis.^60^, ^61^, ^62^ Previous studies termed tumour as ‘hot’ and ‘cold’ according to the tumour response to immunotherapy.^63^ ‘Hot’ tumours have high immunocyte infiltration and activated inflammation while ‘cold’ tumours show the opposite.^64^ In this work, higher infiltration ratio of T cells, NK cells, macrophage and dendritic cell was identified in high riskScore samples than low riskScore samples. It is apparent that high riskScore tumours tend towards a ‘hot’ tumour phenotype, suggesting likely sensitivity to immunotherapy. Other studies have also proved that disordered circadian rhythm was tightly associated with the components of tumour microenvironment, immunocytes activation and immunotherapy reaction.^65^, ^66^, ^67^

In summary, we proved CCGs expression is associated with glioma patient's survival outcome by influencing tumour immune landscape and cell proliferation. The riskScore model based on CCGs can be applied to predict survival outcome and drug sensitivity for tumour patients.

## CONFLICT OF INTEREST

All authors confirm that there are no conflicts of interest.

## AUTHOR CONTRIBUTIONS

ZYW and GHS involved in writing—original draft, formal analysis, investigation and visualization; ZYD, HZ and FF involved in methodology and validation; ZZL and LBZ made formal analysis; NW, FH, MM and NF involved in writing—review and editing; LYZ and QC involved in conceptualization, supervision, project administration and funding acquisition.

## Supporting information

Fig S1

Fig S2

Fig S3

Fig S4

Fig S5

Fig S6

Fig S7

Fig S8

Fig S9

Fig S10

Fig S11

Fig S12

Fig S13

Fig S14

Fig S15

Table S1‐S4

Supplementary Material

## Data Availability

The data for bioinformatic analysis that support the findings of this study are available in TCGA, CGGA and GEO database at https://portal.gdc.cancer.gov, http://www.cgga.org.cn/ and https://www.ncbi.nlm.nih.gov/geo/query/acc.cgi. Data for experiment that support the results of this study are available from the corresponding author upon reasonable request.
