# Supplementary figures and images for: Circadian clock genes promote glioma progression by affecting tumour immune infiltration and tumour cell proliferation
Source: Cell Prolif. 2021 Jan 13;54(3):e12988. doi: 10.1111/cpr.12988 (PMC7941241; doi:10.1111/cpr.12988)

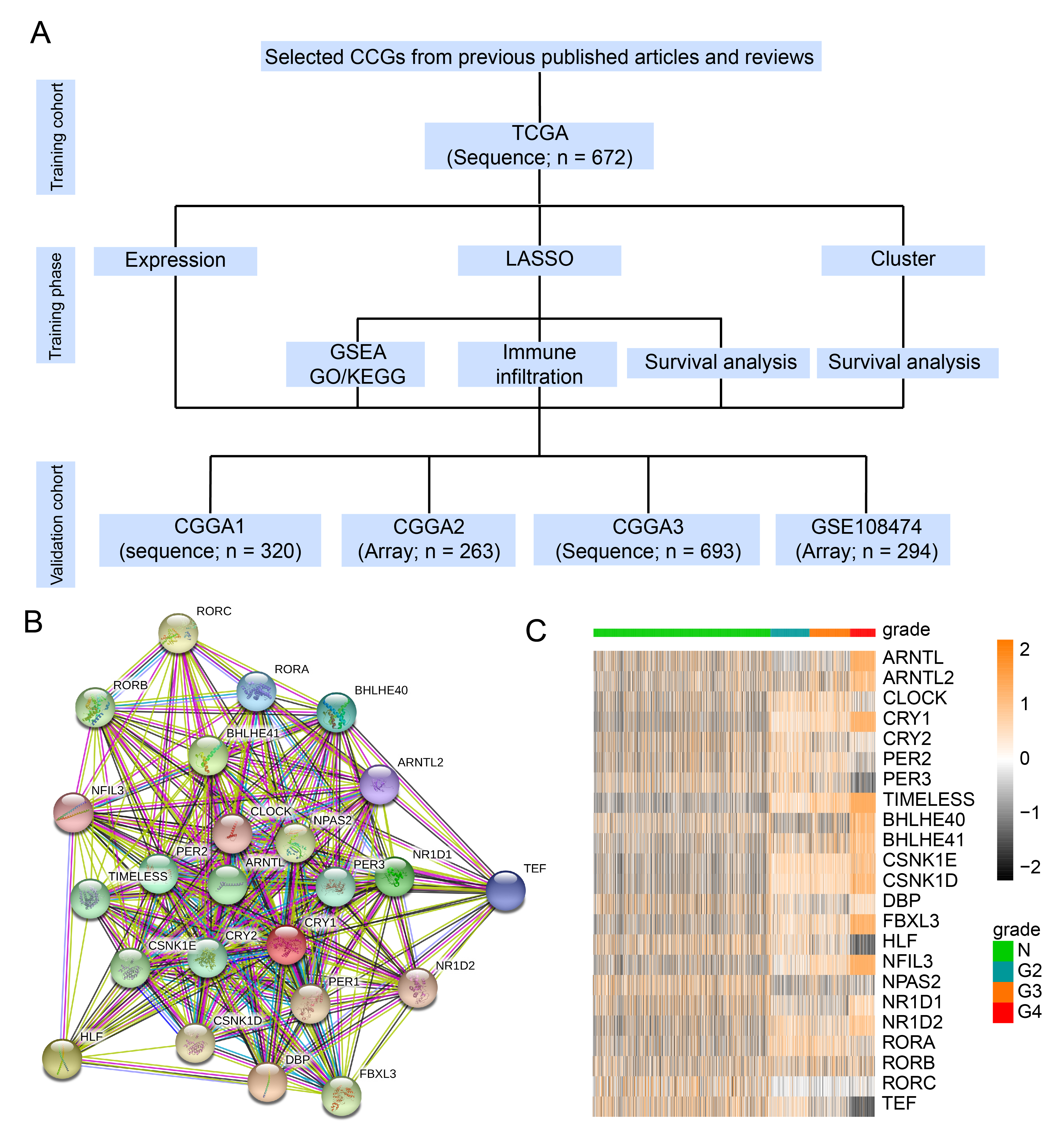

Supplement: Supplementary file 1 — Fig S1 [file CPR-54-e12988-s006.jpg]

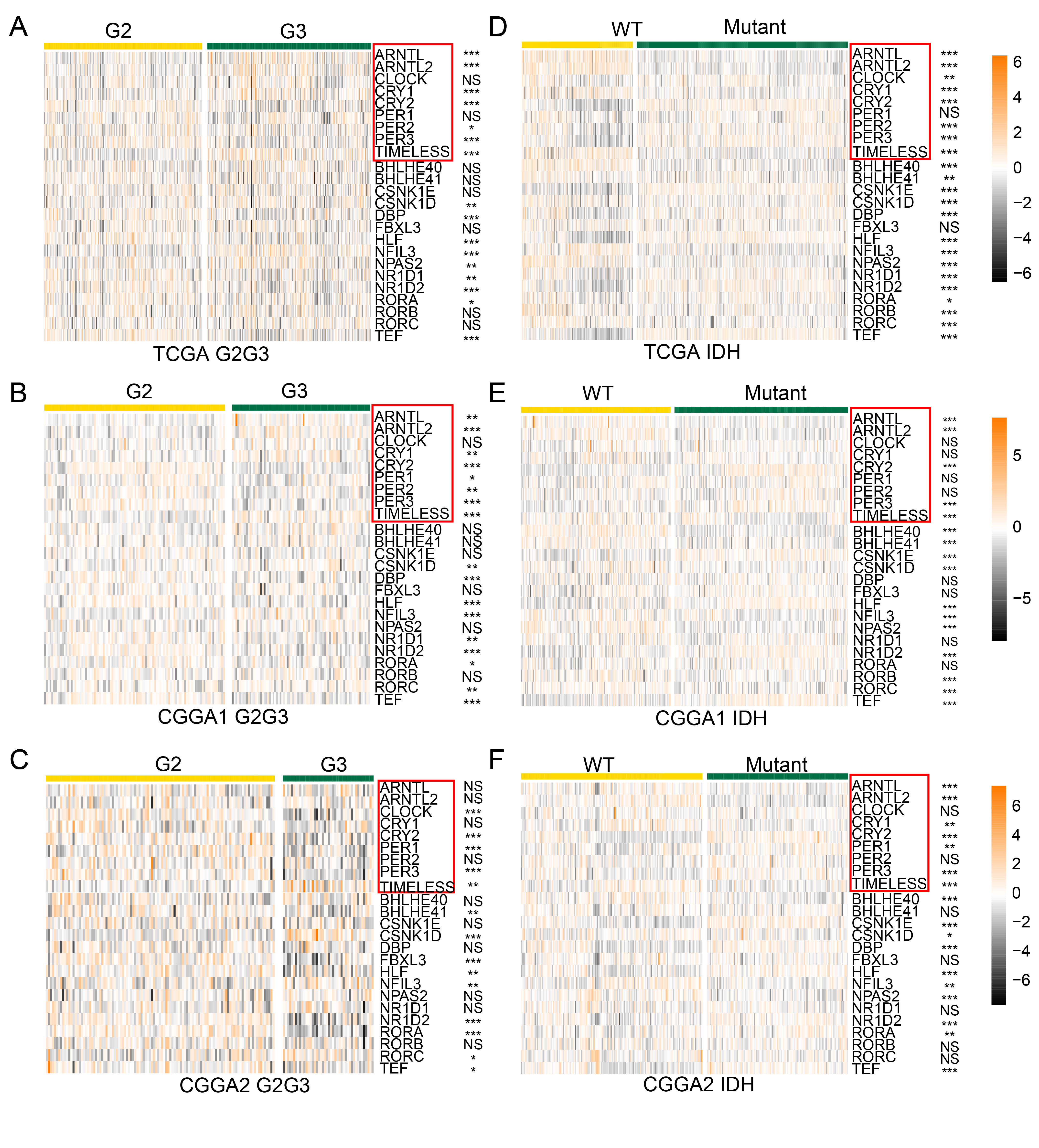

Supplement: Supplementary file 2 — Fig S2 [file CPR-54-e12988-s011.jpg]

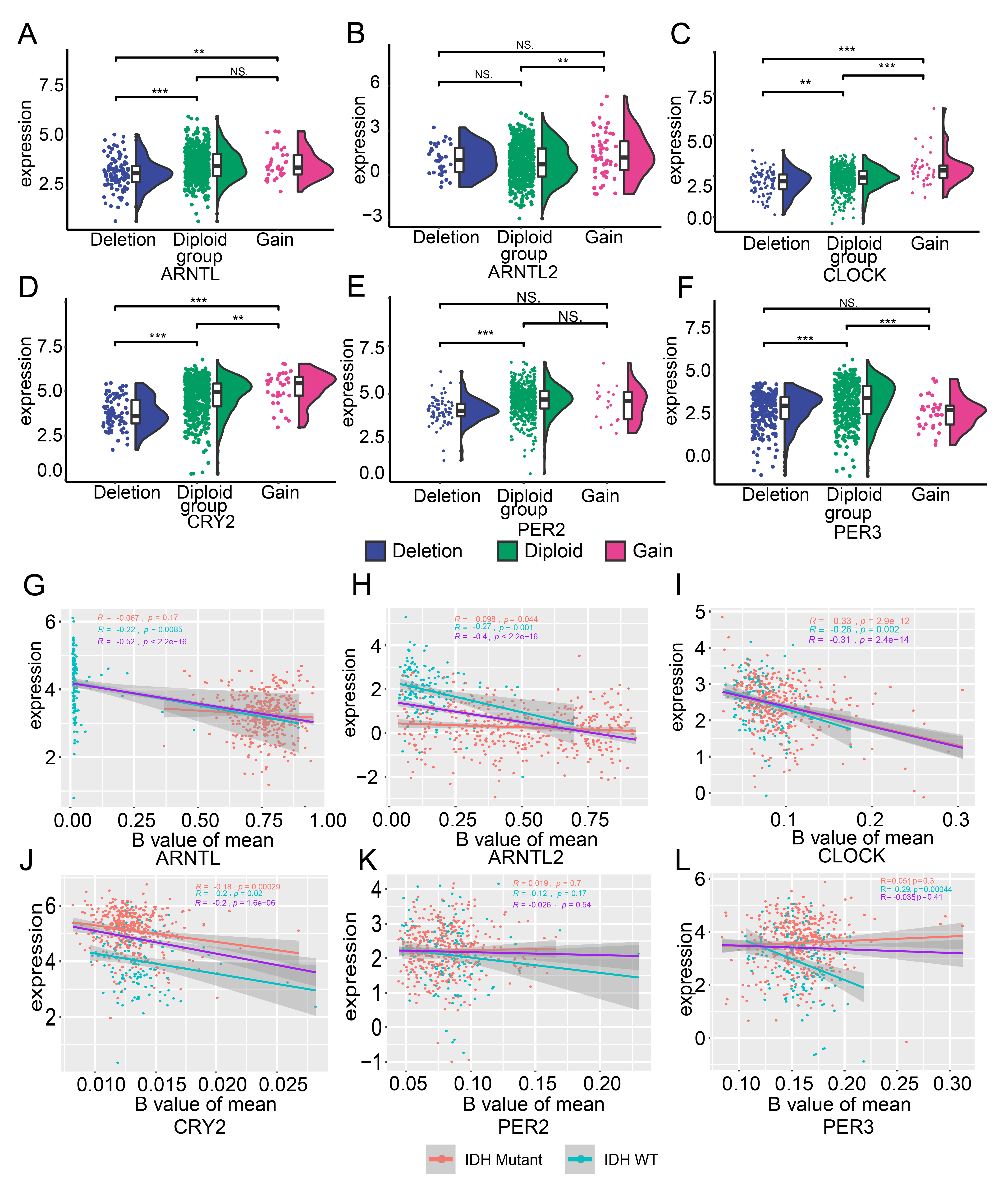

Supplement: Supplementary file 3 — Fig S3 [file CPR-54-e12988-s007.jpg]

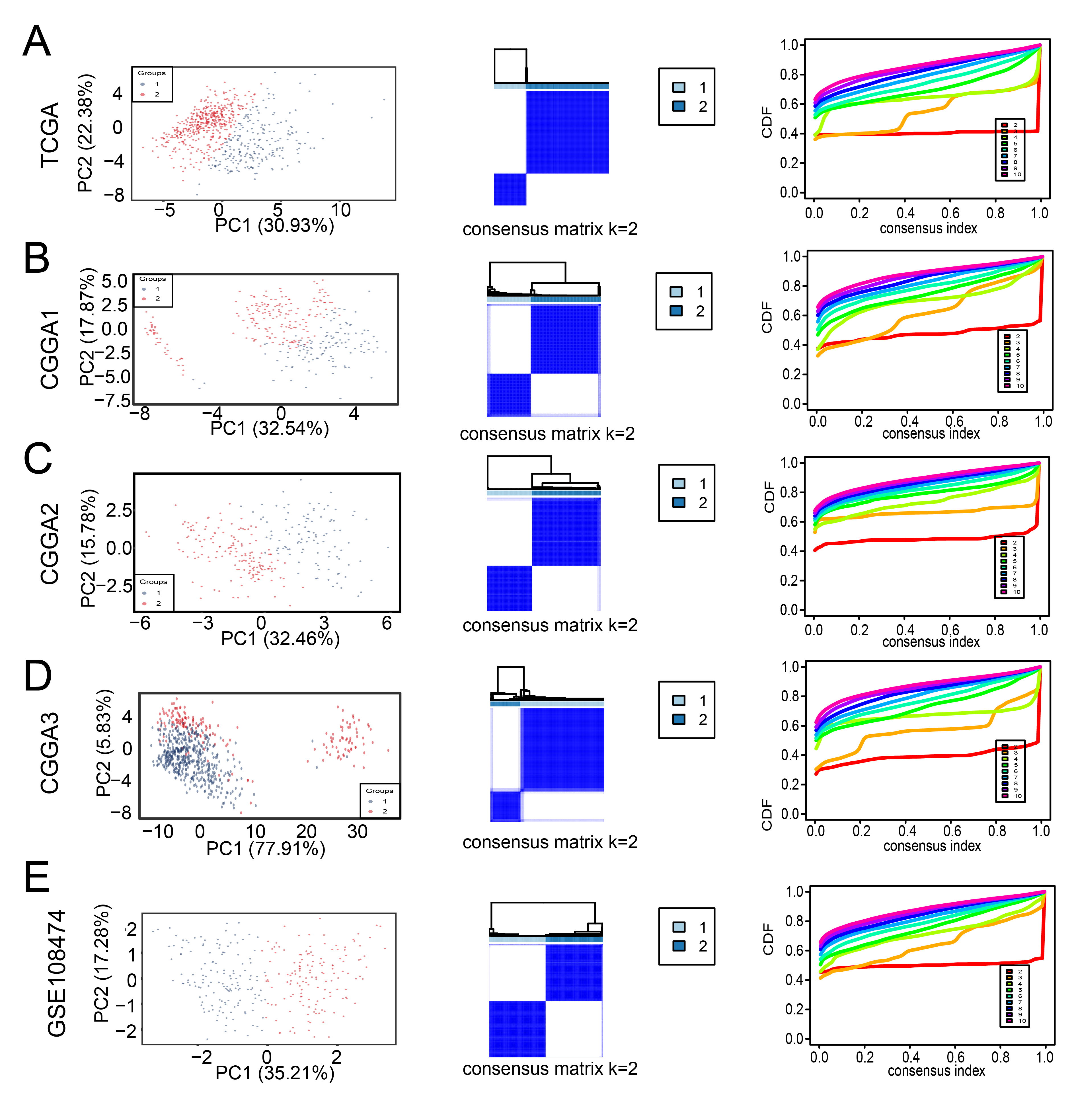

Supplement: Supplementary file 4 — Fig S4 [file CPR-54-e12988-s017.jpg]

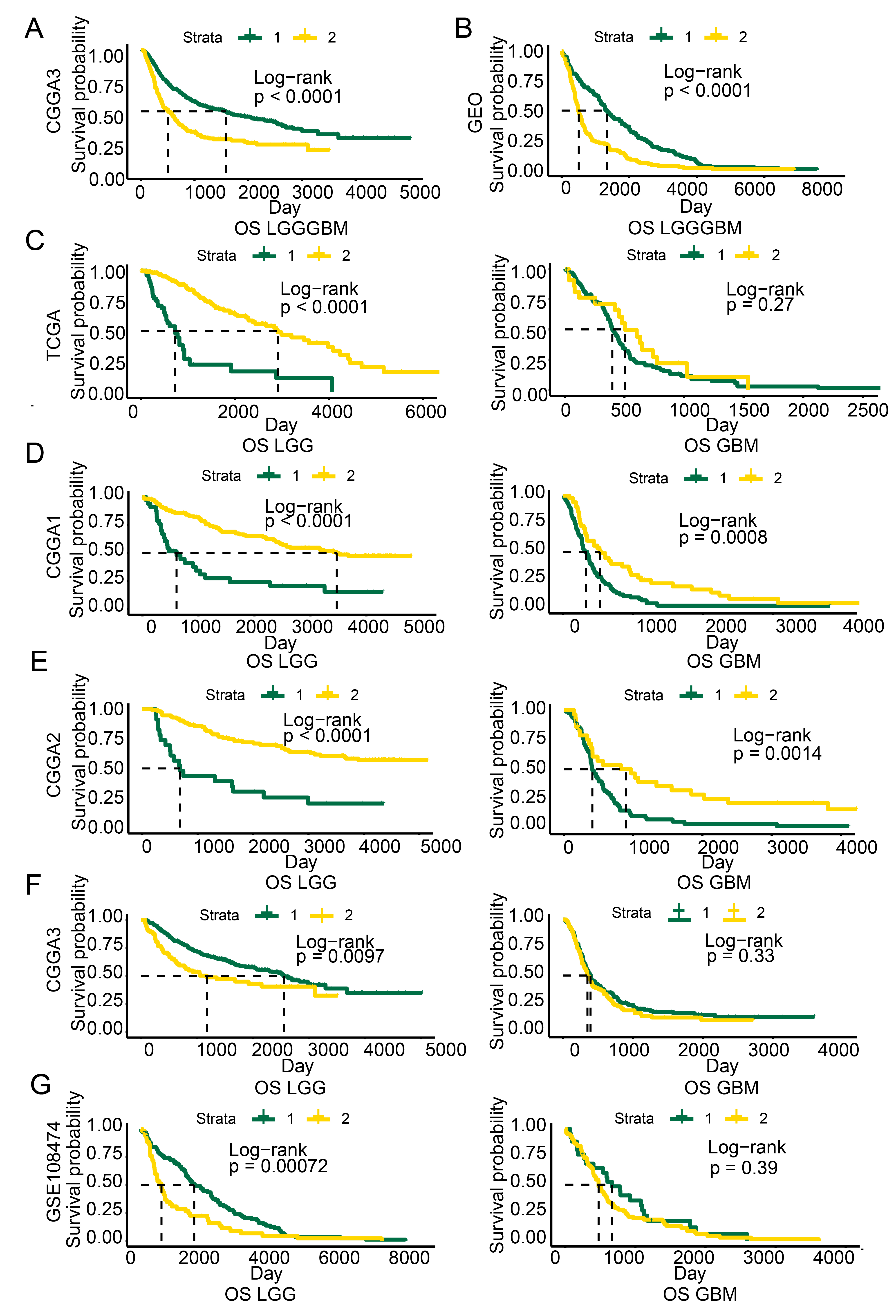

Supplement: Supplementary file 5 — Fig S5 [file CPR-54-e12988-s001.jpg]

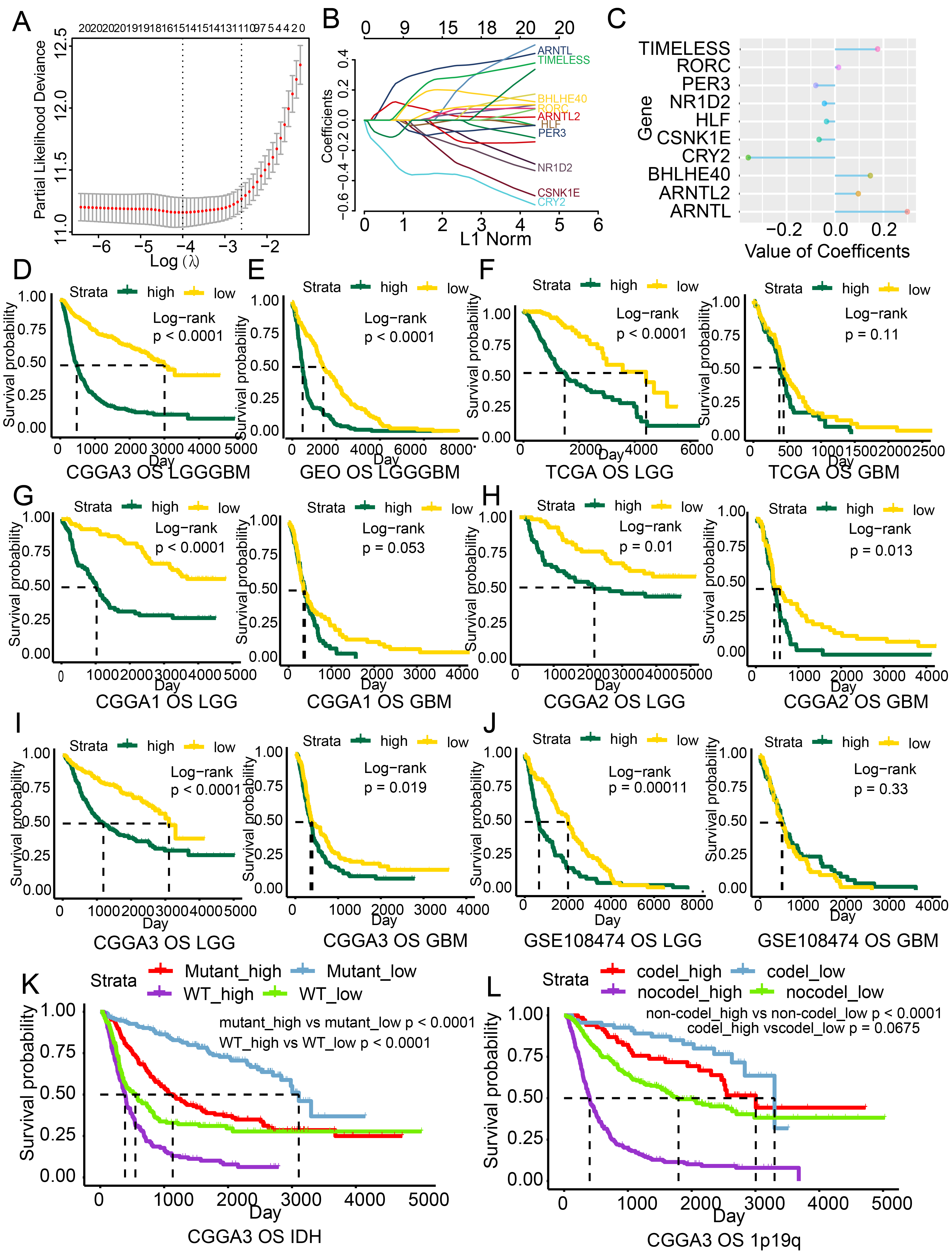

Supplement: Supplementary file 6 — Fig S6 [file CPR-54-e12988-s014.jpg]

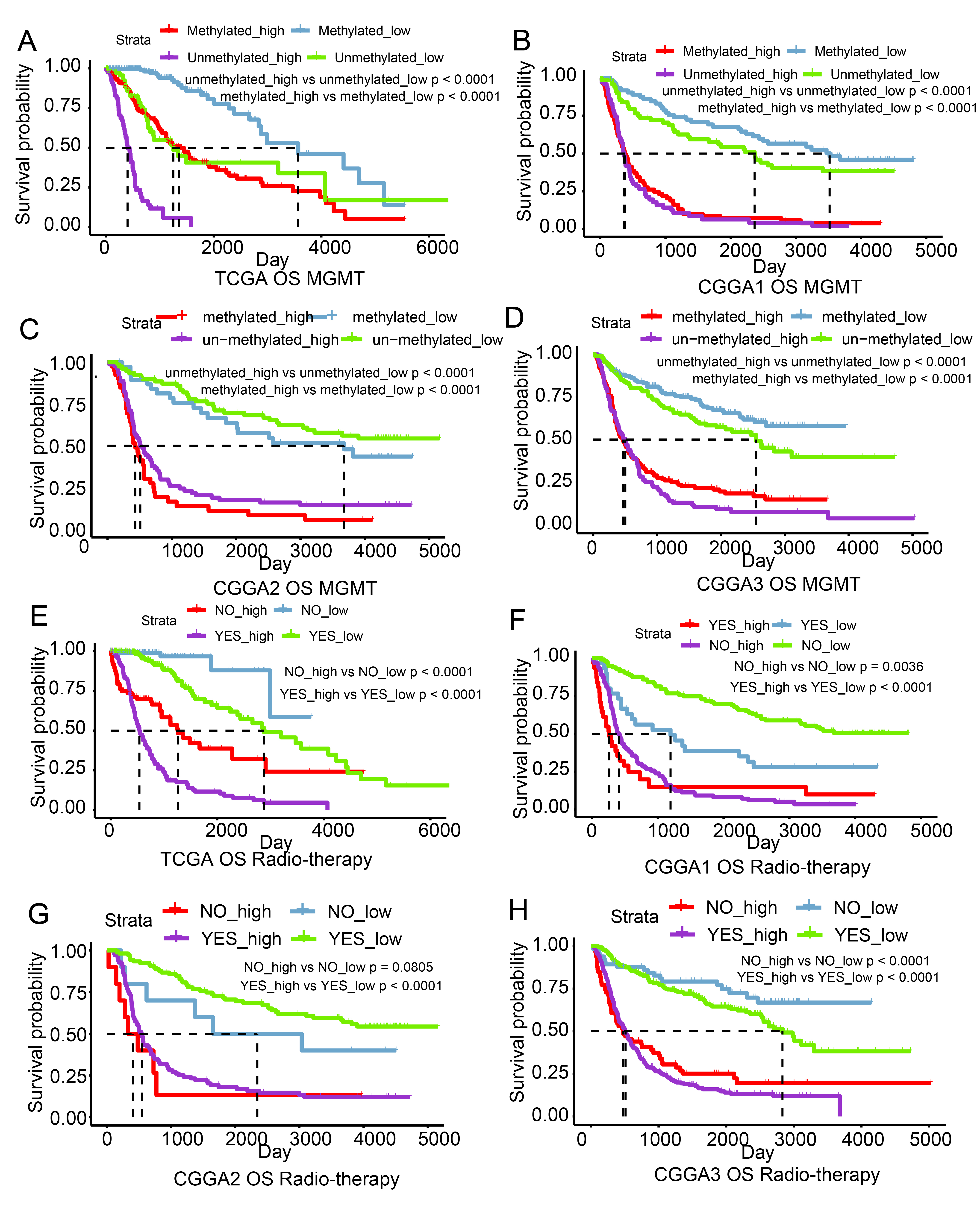

Supplement: Supplementary file 7 — Fig S7 [file CPR-54-e12988-s003.jpg]

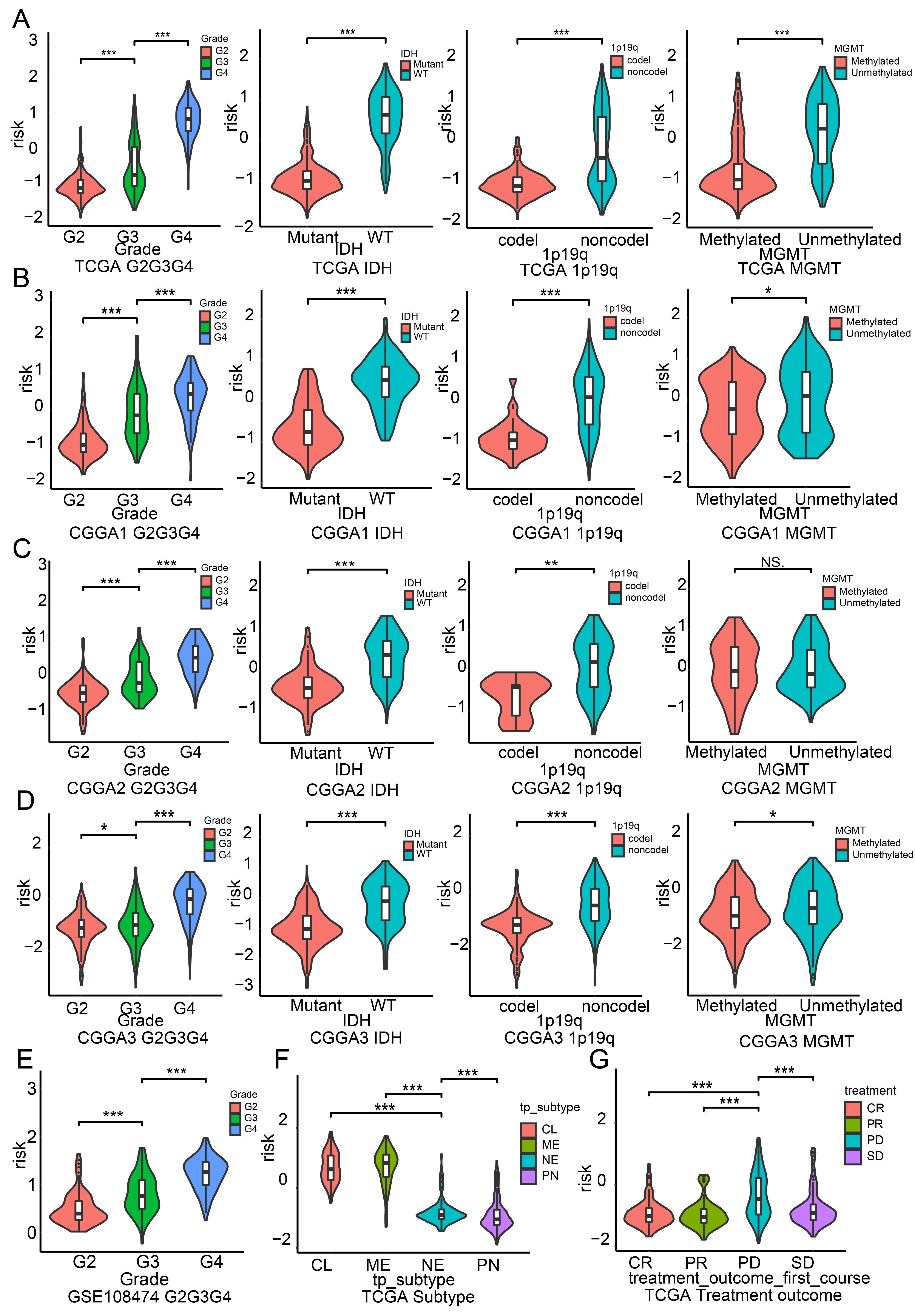

Supplement: Supplementary file 8 — Fig S8 [file CPR-54-e12988-s004.jpg]

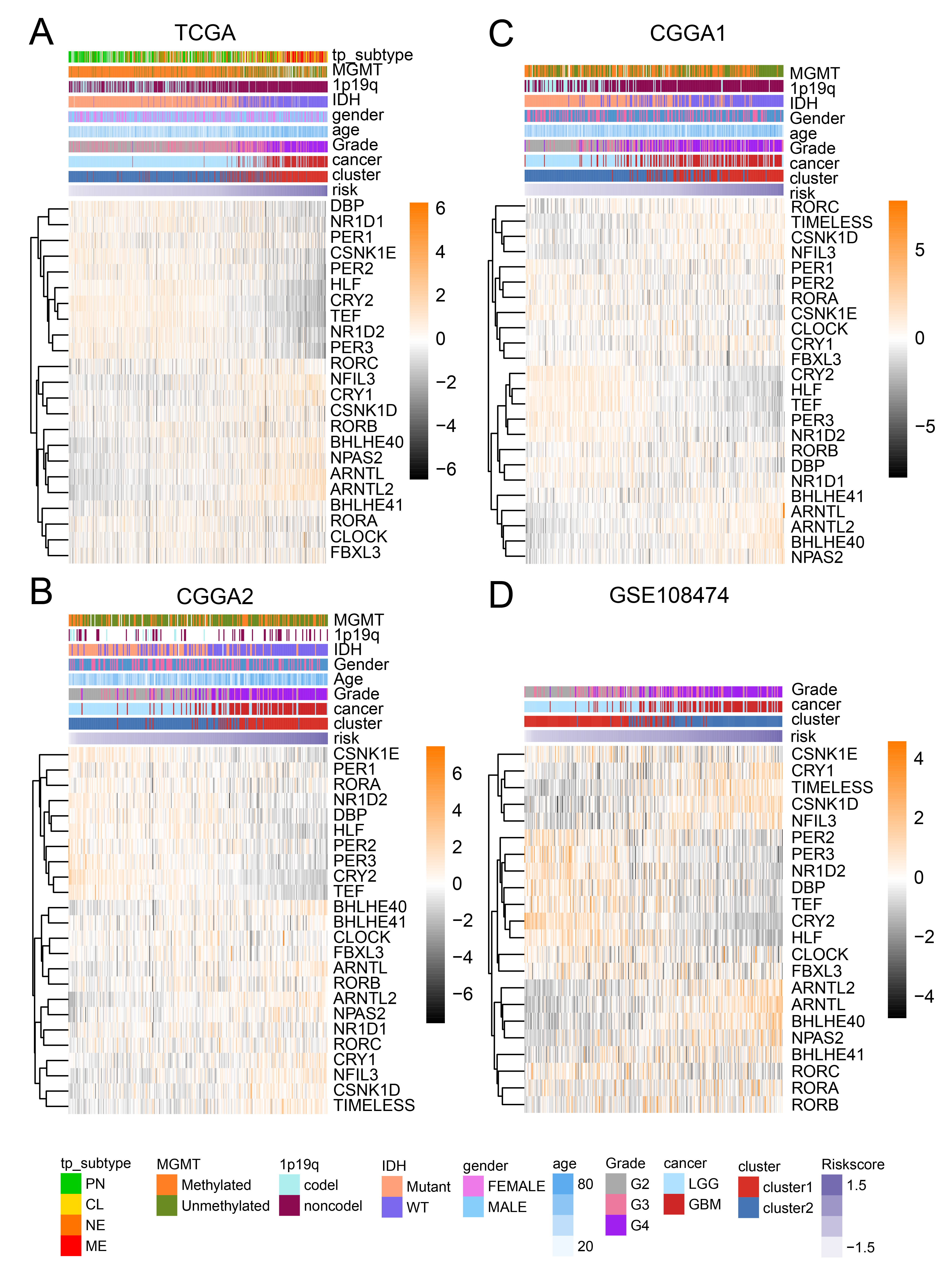

Supplement: Supplementary file 9 — Fig S9 [file CPR-54-e12988-s013.jpg]

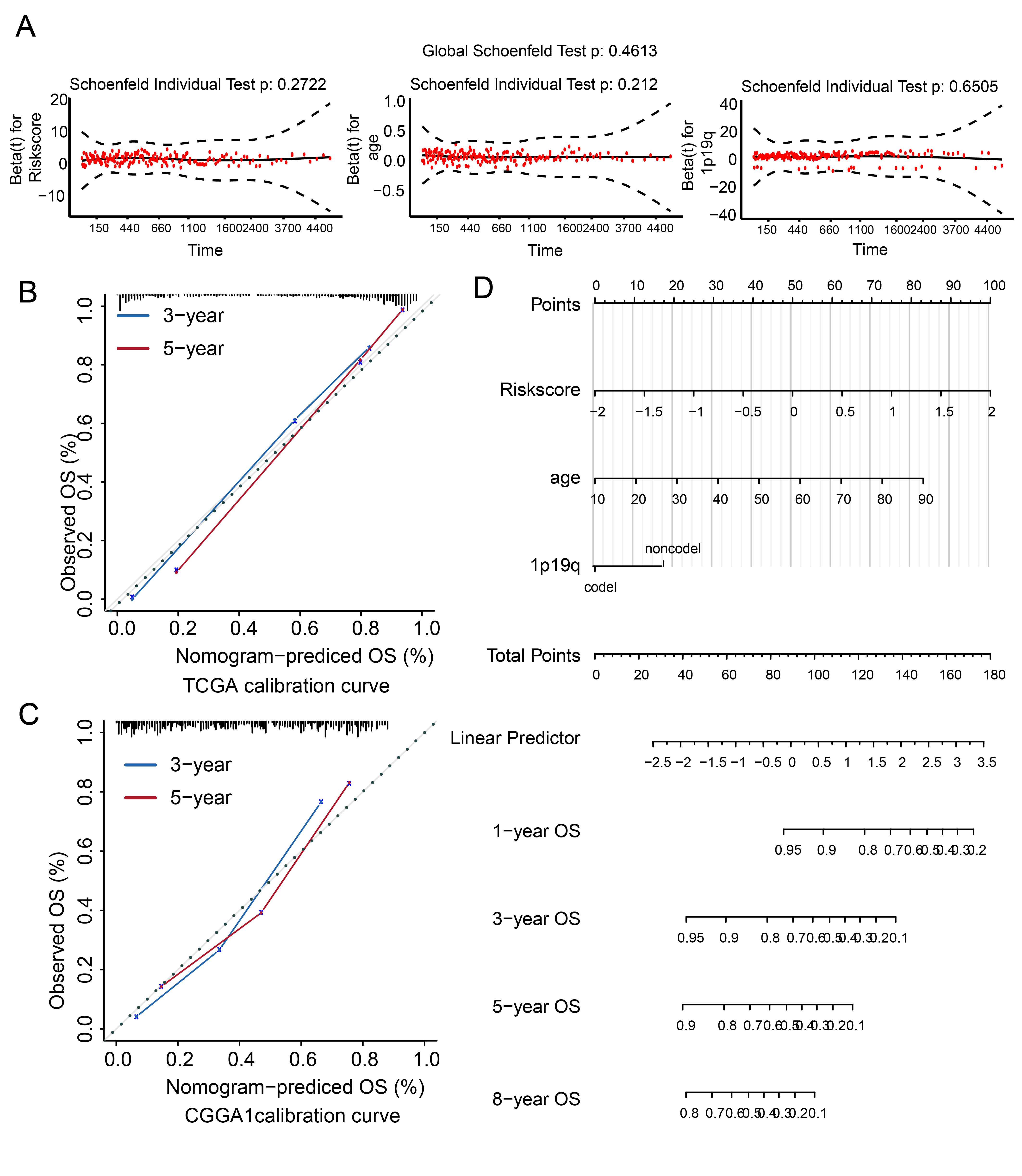

Supplement: Supplementary file 10 — Fig S10 [file CPR-54-e12988-s008.jpg]

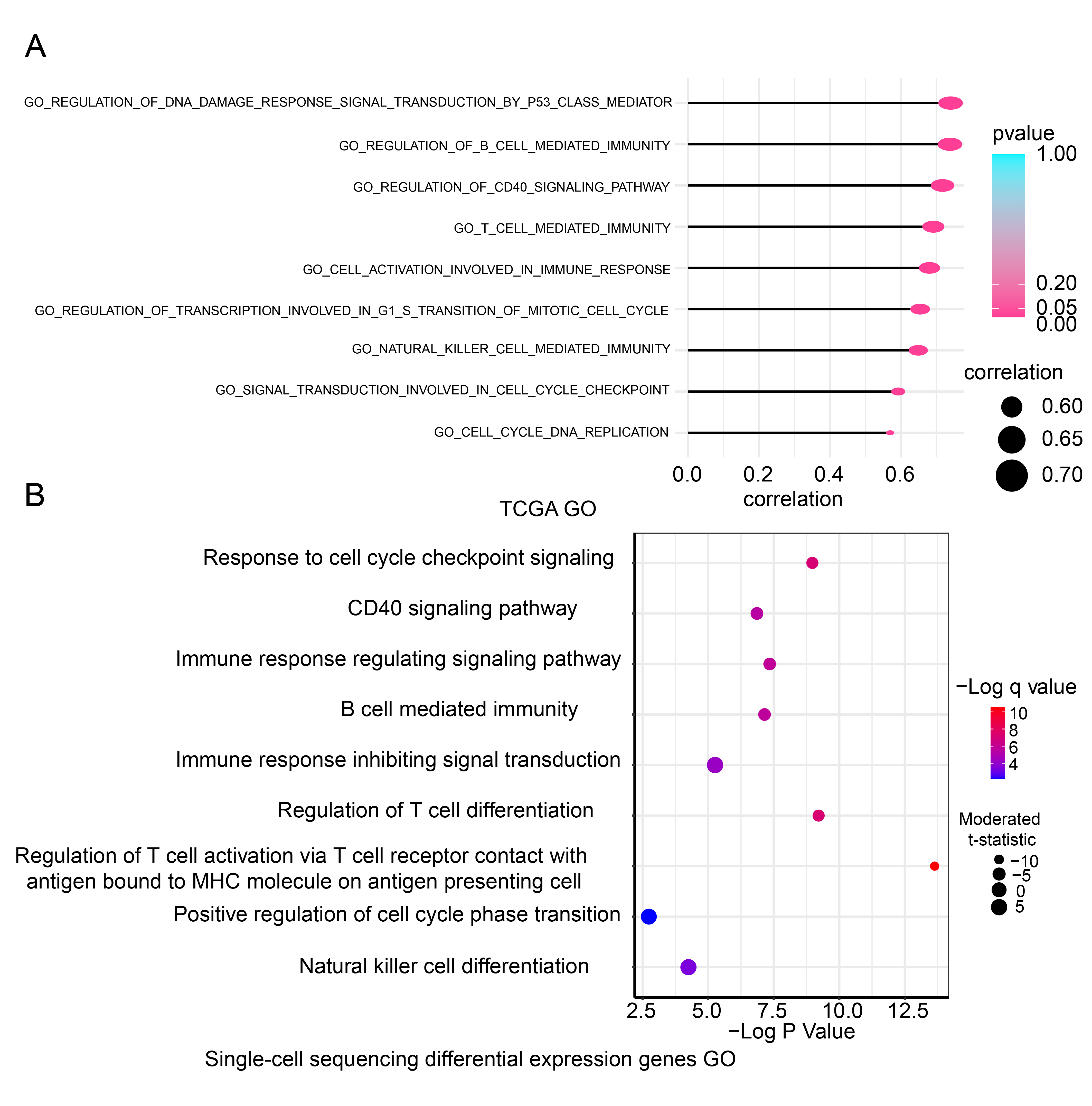

Supplement: Supplementary file 11 — Fig S11 [file CPR-54-e12988-s015.jpg]

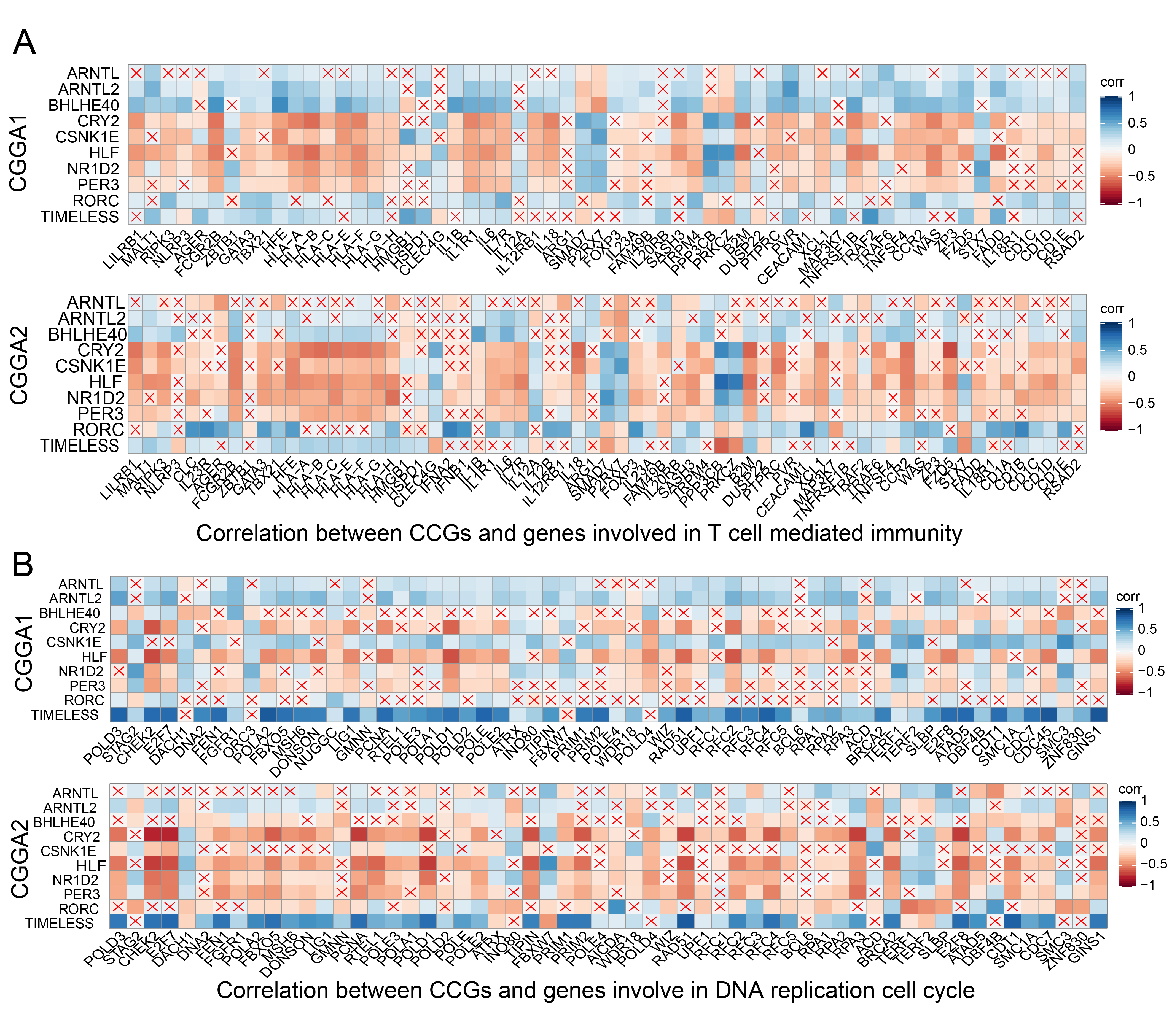

Supplement: Supplementary file 12 — Fig S12 [file CPR-54-e12988-s016.jpg]

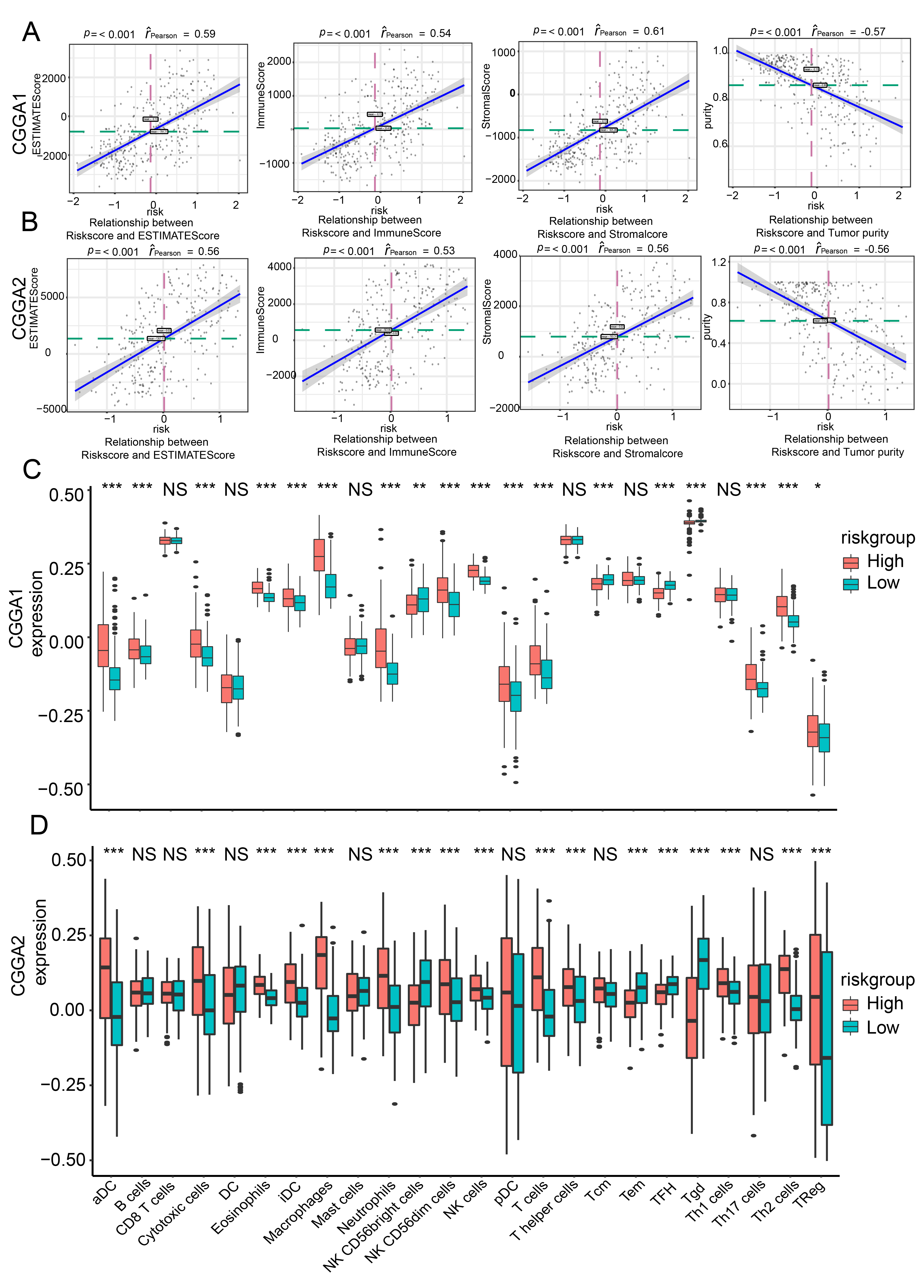

Supplement: Supplementary file 13 — Fig S13 [file CPR-54-e12988-s005.jpg]

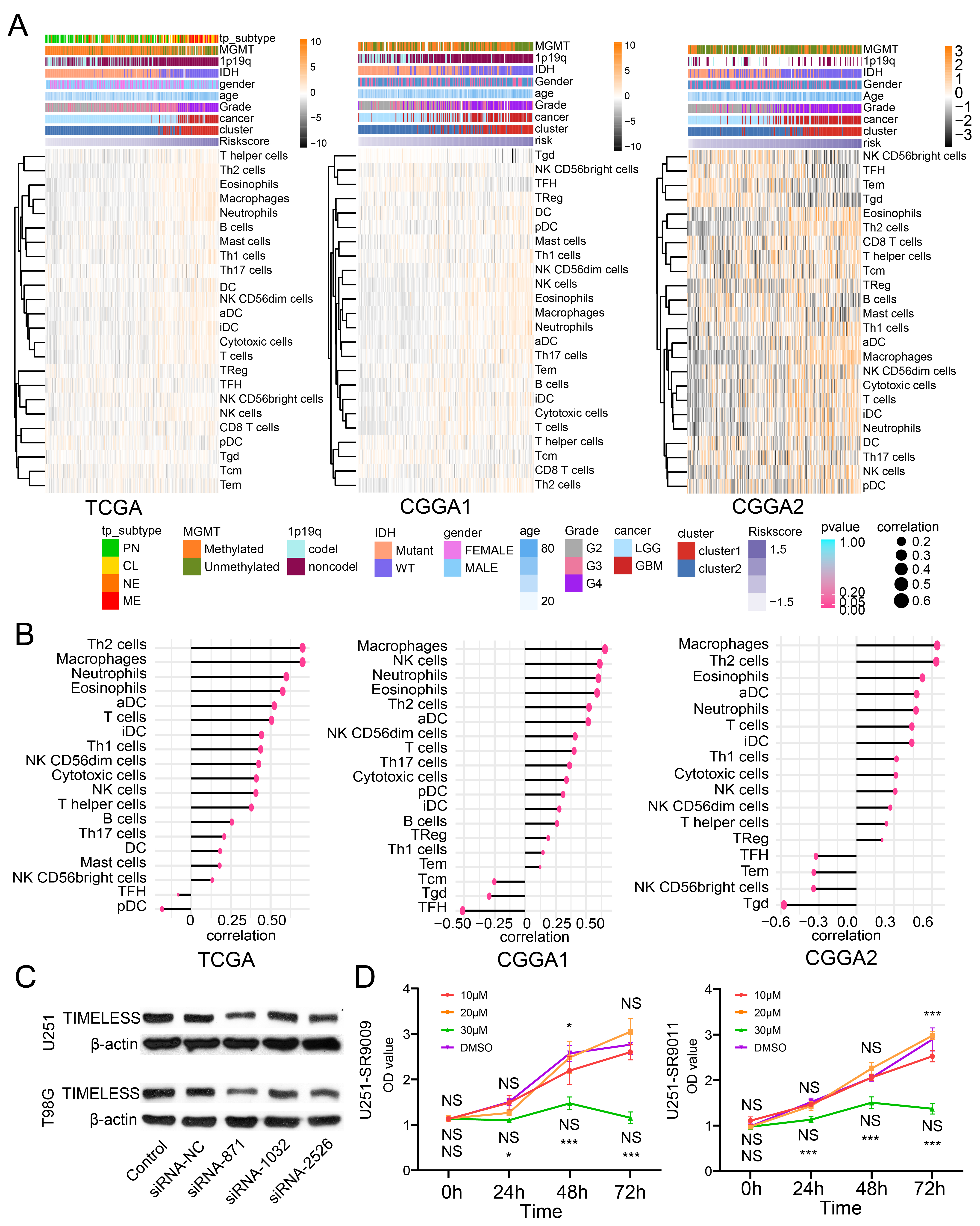

Supplement: Supplementary file 14 — Fig S14 [file CPR-54-e12988-s010.jpg]

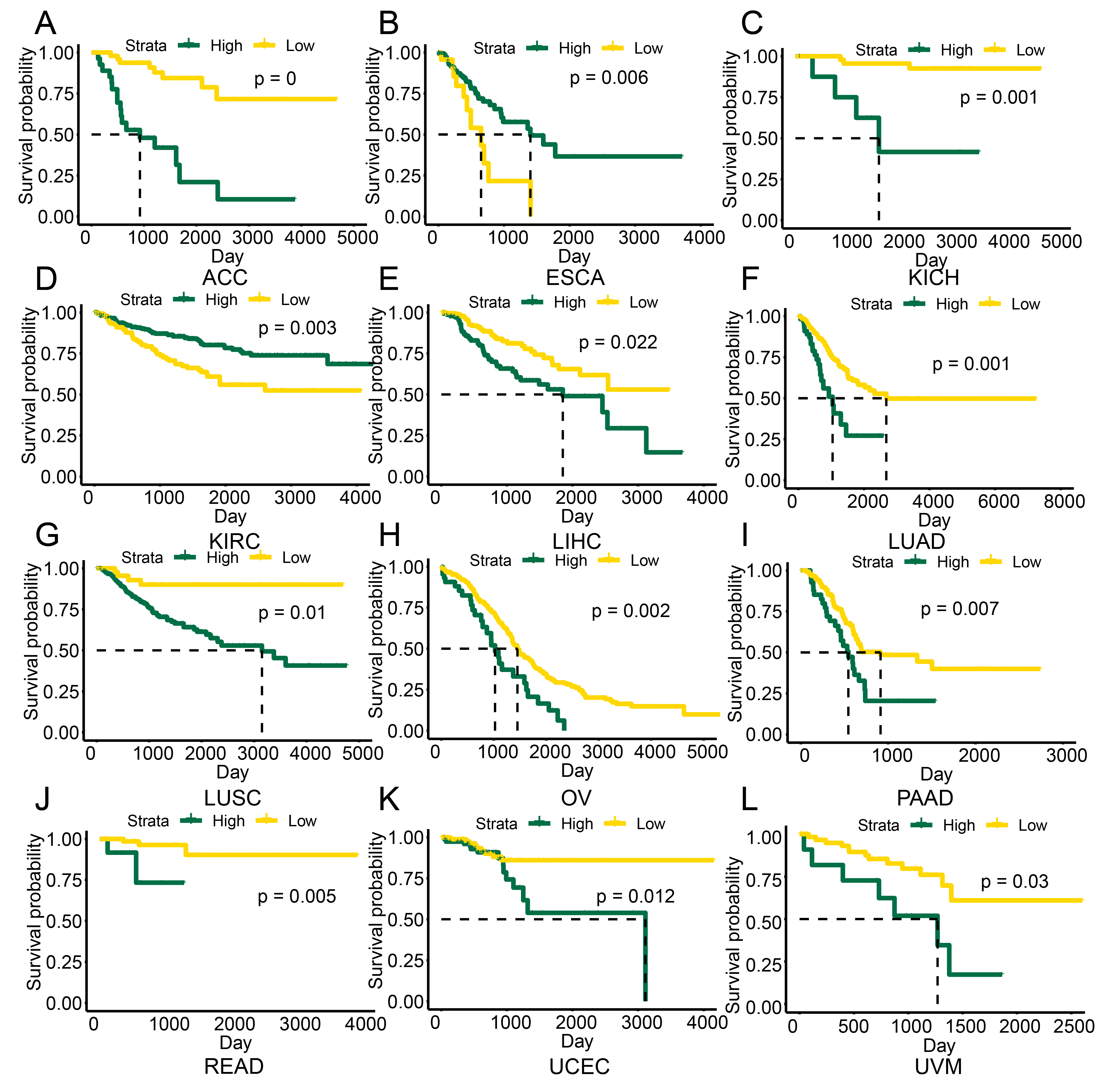

Supplement: Supplementary file 15 — Fig S15 [file CPR-54-e12988-s002.jpg]
