# Supplementary material for: Circadian clock genes promote glioma progression by affecting tumour immune infiltration and tumour cell proliferation
Source: Cell Prolif. 2021 Jan 13;54(3):e12988. doi: 10.1111/cpr.12988 (PMC7941241; doi:10.1111/cpr.12988)
Supplement: Supplementary file 16 — Table S1‐S4 [file CPR-54-e12988-s012.docx]

| Details | TCGA | CGGA1 | CGGA2 | CGGA3 | GEO |
| --- | --- | --- | --- | --- | --- |
| Number of samples | 672 | 320 | 263 | 693 | 294 |
| Cancer (LGG : GBM) | 522:150 | 180:136 | 108:155 | 443:249 | 170:124 |
| Grade (G2 : G3 : G4) | 256:265:150 | 102:78:136 | 111:44:108 | 188:255:249 | 86:82:124 |
| Age (≥45 : <45) | 329:43 | 132:188 | 114:147 | 310:382 | \ |
| Gender (Female : Male) | 285:387 | 121:199 | 106:157 | 295:398 | \ |
| MGMT (Methylated : Unmethylated) | 478:157 | 154:147 | 80:170 | 315:227 | \ |
| 1p19q (Codel : Non-codel) | 171:497 | 67:250 | 14:50 | 145:478 | \ |
| IDH (Wildtype : Mutant) | 228:434 | 148:172 | 148:113 | 356:286 | \ |
| Radio-therapy (Yes : No) | 415:203 | 241:65 | 224:27 | 510:136 | \ |
| Chemo-therapy (Yes : No) | \ | 189:110 | 110:133 | 486:161 | \ |

**Supplementary Table 1 |** Information about the TCGA cohort, CGGA1 cohort, CGGA2 cohort, CGGA3 cohort, and GEO cohort. (LGG: Lower Grade Glioma; GBM: Glioblastoma; G2: WHO II; G3: WHO III; G4: WHO IV)

| Gene | Hazard ratio | 95%CI | P value |
| --- | --- | --- | --- |
| ARNTL | 2.622 | 2.219-3.099 | 1.08E-29 |
| ARNTL2 | 1.883 | 1.702-2.082 | 9.72E-35 |
| BHLHE40 | 1.573 | 1.407-1.76 | 1.98E-15 |
| BHLHE41 | 1.066 | 0.952-1.194 | 0.269 |
| CLOCK | 0.795 | 0.667-0.948 | 0.010519 |
| CRY1 | 2.084 | 1.672-2.598 | 6.31E-11 |
| CRY2 | 0.496 | 0.45-0.546 | 5.79E-46 |
| CSNK1E | 0.46 | 0.399-0.53 | 6.95E-27 |
| CSNK1D | 1.472 | 1.058-2.048 | 0.021762 |
| DBP | 0.508 | 0.433-0.596 | 1.03E-16 |
| FBXL3 | 1.069 | 0.819-1.396 | 0.621321 |
| HLF | 0.572 | 0.526-0.623 | 1.85E-38 |
| NFIL3 | 2.177 | 1.867-2.538 | 2.89E-23 |
| NPAS2 | 1.436 | 1.274-1.618 | 2.89E-09 |
| NR1D1 | 0.667 | 0.583-0.763 | 3.77E-09 |
| NR1D2 | 0.463 | 0.404-0.531 | 5.37E-28 |
| PER1 | 0.891 | 0.785-1.011 | 0.072784 |
| PER2 | 0.607 | 0.525-0.703 | 2.15E-11 |
| PER3 | 0.584 | 0.532-0.642 | 3.67E-29 |
| RORA | 0.74 | 0.639-0.858 | 6.75E-05 |
| RORB | 1.078 | 0.995-1.167 | 0.064796 |
| RORC | 1.23 | 1.138-1.329 | 1.75E-07 |
| TEF | 0.49 | 0.443-0.542 | 7.48E-44 |
| TIMELESS | 1.779 | 1.591-1.99 | 5.67E-24 |

**Supplementary Table 2 |** Univariate Cox analysis of CCGs.

|  | Univariate Cox | | Multivariate Cox | |
| --- | --- | --- | --- | --- |
| Factors | P value | Hazard ratio (95% CI) | P value | Hazard ratio (95% CI) |
| Riskscore | < 0.001 | 3.985(3.305−4.806) | < 0.001 | 2.331(1.546−3.516) |
| WHO III | < 0.001 | 3.646(2.390−5.562) | 0.009 | 1.857(1.167−2.955) |
| WHO IV | < 0.001 | 20.656(12.828−33.262) | 0.089 | 1.838(0.911−3.706) |
| Age | < 0.001 | 1.062(1.050−1.074) | < 0.001 | 1.040(1.026−1.054) |
| IDH | < 0.001 | 7.907(5.748−10.878) | 0.398 | 1.319(0.694−2.508) |
| 1p19q | < 0.001 | 3.995(2.529−6.309) | 0.033 | 1.810(1.049−3.123) |
| MGMT | < 0.001 | 3.181(2.344−4.318) | 0.354 | 1.198(0.817−1.758) |
| Mesenchymal | 0.451 | 1.166(0.782−1.739) | 0.170 | 1.344(0.881−2.049) |
| Neural | < 0.001 | 0.174(0.105−0.287) | 0.247 | 1.517(0.750−3.069) |
| Proneural | < 0.001 | 0.156(0.104−0.234) | 0.229 | 1.488(0.779−2.842) |

**Supplementary Table 3 |** Univariate Cox analysis and Multivariate Cox analysis based on the TCGA dataset.

| Drugs | Enrichment score | P-value |
| --- | --- | --- |
| podophyllotoxin | -0.92 | 8.00E-05 |
| nystatin | -0.901 | 0.0018 |
| amantadine | -0.778 | 0.00507 |
| Prestwick-857 | -0.72 | 0.01241 |
| thiamine | -0.711 | 0.04927 |
| Trolox C | -0.701 | 0.01667 |
| josamycin | -0.697 | 0.00555 |
| diprophylline | -0.679 | 0.00787 |
| atractyloside | -0.677 | 0.00813 |
| chlorhexidine | -0.673 | 0.00879 |
| cinchonine | -0.666 | 0.02829 |
| memantine | -0.624 | 0.04979 |
| diethylstilbestrol | -0.62 | 0.00963 |
| sodium phenylbutyrate | -0.575 | 0.0103 |
| PNU-0230031 | 0.454 | 0.04898 |
| PNU-0251126 | 0.574 | 0.02207 |
| isradipine | 0.627 | 0.04844 |
| epiandrosterone | 0.627 | 0.0485 |
| mesoridazine | 0.629 | 0.04752 |
| 6-bromoindirubin-3'-oxime | 0.641 | 0.00232 |
| chlorambucil | 0.644 | 0.03867 |
| amrinone | 0.65 | 0.03553 |
| simvastatin | 0.669 | 0.02678 |
| nimesulide | 0.674 | 0.02495 |
| tremorine | 0.677 | 0.02379 |
| xylometazoline | 0.686 | 0.02085 |
| parthenolide | 0.705 | 0.0157 |
| methazolamide | 0.783 | 0.00414 |
| talampicillin | 0.827 | 0.00143 |
| ticarcillin | 0.846 | 0.00703 |
| piperlongumine | 0.855 | 0.04277 |
| naftopidil | 0.896 | 0.00216 |
| cromoglicic acid | 0.911 | 0.01606 |
| withaferin A | 0.917 | 4.00E-05 |
| STOCK1N-35874 | 0.932 | 0.00873 |
| 5182598 | 0.992 | 6.00E-05 |

**Supplementary Table 4 |** Drugs prediction from CMap.
