# Supplementary material for: Circadian clock genes promote glioma progression by affecting tumour immune infiltration and tumour cell proliferation
Source: Cell Prolif. 2021 Jan 13;54(3):e12988. doi: 10.1111/cpr.12988 (PMC7941241; doi:10.1111/cpr.12988)
Supplement: Supplementary file 17 — Supplementary Material [file CPR-54-e12988-s009.docx]

**Supplementary Figure 1 |** Flow chart and correlation between CCGs. (A) Flow chart summarizing the study design. (B) Protein-protein interaction of CCGs. (C) Expression profile of CCGs in normal tissue and glioma.

**Supplementary Figure 2** | Expression profile of CCGs. Heatmap of CCG expression differences between grade II and grade III glioma from the TCGA (A), CGGA1 (B), and CGGA2 (C) datasets. Expression profile based on sample IDH status from the TCGA (D), CGGA1 (E), and CGGA2 (F) datasets. The core clock genes are labelled with red. NS: not statistically significant; *p<0.05; **p<0.01; ***p<0.001.

**Supplementary Figure 3 |** CNVs and DNA methylation of CCGs. CNVs for ARNTL (A), ARNTL2 (B), CLOCK (C), CRY2 (D), PER2 (E), PER3 (F). DNA methylation status for ARNTL (G), ARNTL2 (H), CLOCK (I), CRY (J), PER2 (K) and PER3 (L) along with IDH status. NS: not statistically significant; *p<0.05; **p<0.01; ***p<0.001.

**Supplementary Figure 4 |** The cluster model based on CCGs. Principal components analysis of the cluster model using the TCGA (A), CGGA1 (B), CGGA2 (C), CGGA3 (D), and GSE108474 (E) datasets.

**Supplementary Figure 5 |** Survival analysis based on the cluster model. Survival analysis of the cluster model based on the LGGGBM cohort from the CGGA3 (A, P-value < 0.0001) and GSE108474 (B, P-value < 0.0001) datasets. Cluster model-based survival outcomes for LGG and GBM (C: TCGA, LGG: P-value < 0.0001,GBM: P-value = 0.27; D: CGGA1, LGG: P-value < 0.0001, GBM: P-value = 0.0006; E: CGGA2, LGG: P-value < 0.0001, GBM: P-value = 0.0014; F: CGGA3, LGG: P-value < 0.0001, GBM: P-value = 0.33; G: GSE108474, LGG: P-value < 0.0001, GBM: P-value = 0.39).

**Supplementary Figure 6 |** The LASSO analysis based on CCGs. (A-C) Regression coefficient diagram of the LASSO regression analysis. Survival analysis of the riskScore model based on the LGGGBM cohort from the CGGA3 (D, P-value < 0.0001) and GSE108474 (E, P-value < 0.0001) datasets. The riskScore model-based survival outcome differences for LGG and GBM in the TCGA (F, LGG P-value < 0.0001; GBM: P-value = 0.11), CGGA1 (G, LGG: P-value < 0.0001; GBM: P-value = 0.053), CGGA2 (H, LGG: P-value = 0.01; GBM: P-value = 0.013), CGGA3 (I, LGG: P-value < 0.0001; GBM: P-value = 0.019) and GSE108474 (J, LGG: P-value = 0.0011; GBM: P-value = 0.33) dataset. Overall survival analysis of subgroup based on IDH status (K, mutant_high vs mutant_low < 0.0001, WT_high vs WT_low < 0.0001) and 1p19q status (L, noncodel_high vs noncodel_low < 0.0001, codel_high vs codel_low = 0.0675) from the CGGA3 datatset.

**Supplementary Figure 7 |** Survival analysis of subgroups based on the riskScore model in the TCGA and CGGA dataset. MGMT status (TCGA: A, methylated_high vs methylated_low < 0.0001, unmethylated_high vs unmethylated_low < 0.0001; CGGA1: B, methylated_high vs methylated_low < 0.0001, unmethylated_high vs unmethylated_low < 0.0001; CGGA2: C, methylated_high vs methylated_low < 0.0001, unmethylated_high vs unmethylated_low < 0.0001; CGGA3: D, methylated_high vs methylated_low < 0.0001, unmethylated_high vs unmethylated_low < 0.0001). Rradio-therapy status (TCGA: E, no_high vs no_low < 0.0001, yes_high vs yes_low < 0.0001; CGGA1: F, no_high vs no_low = 0.0036, yes_high vs yes_low < 0.0001; CGGA2: G, no_high vs no_low = 0.0805, yes_high vs yes_low < 0.0001; CGGA3: H, no_high vs no_low < 0.0001, yes_high vs yes_low < 0.0001).

**Supplementary Figure 8 |** The relationship between riskScore and clinical features. The riskScore distribution in tumor grade, IDH status, 1p19q status and MGMT status in the TCGA (A), CGGA1 (B), CGGA2 (C), CGGA3 (D) dataset. (E) Association between riskScore and tumor grade in the GSE108474 dataset. (F) Relationship between riskScore and tumor subtypes from the TCGA dataset. (G) RiskScore is associated with treatment outcome in the TCGA dataset. CL: classical, ME: mesenchymal, NE: neural, PN: proneural, CR: complete remission/response, PR: partial remission/response, PD: progressive disease, SD: stable disease. NS: not statistically significant; *p<0.05; **p<0.01; ***p<0.001.

**Supplementary Figure 9 |** Expression profile of CCGs and its potential biofunction. Heatmap of the CCG expression profile and corresponding clinical features based on the riskScore from the TCGA (A), CGGA1 (B), CGGA2 (C) and GSE108474 (D) datasets.

**Supplementary Figure 10 |** The construction of nomogram. (A) Schoenfeld individual test of variables, including riskScore (p: 0.2722), age (p: 0.212) and 1p19q (p: 0.6505), included in the nomogram. Calibration curve of 3-year (blue) and 5-year (red) overall survival based on the TCGA (B) and CGGA1 (C) datasets. (D) Nomogram based on riskScore, age and 1p19q status.

**Supplementary Figure 11 |** Correlation of pathways from the GO enrichment analysis based on the TCGA dataset (A) and single-cell sequencing analysis (B).

**Supplementary Figure 12 |** Correlation between core CCGs and genes involved in T cell mediated immunity and cell cycle. Correlation between riskScore related genes and genes related to T cell mediated immunity (A) and DNA replication cell cycle (B) according to the CGGA1 and CGGA2 datasets.

**Supplementary Figure 13 |** Immunocytes infiltration in the CGGA dataset. Expression of infiltrated immunocytes from the CGGA1 (A) and CGGA2 (B) datasets. Correlation of riskScore and ESTIMATE score (CGGA1: r = 0.59, P-value < 0.001; CGGA2: r = 0.56, P-value < 0.001), immune score (CGGA1: r = 0.54, P-value < 0.001; CGGA2: r = 0.53, P-value < 0.001), stromal score (CGGA1: r = 0.61, P-value < 0.001; CGGA2: r = 0.56, P-value < 0.001), and tumor purity (CGGA1: r = -0.57, P-value < 0.001; CGGA2: r = -0.56, P-value < 0.001). The expression profile of immunocytes in the CGGA1 (C) and CGGA2 (D) dataset. NS: not statistically significant; *p<0.05; **p<0.01; ***p<0.001.

**Supplementary Figure 14 |** Immunocytes infiltration in the TCGA and CGGA dataset. (A) Expression profile of infiltrated immunocytes and clinical features based on the riskScore from the TCGA, CGGA1, and CGGA2 datasets. (B) Correlation of infiltrated immunocytes according to riskScore from the TCGA, CGGA1, and CGGA2 datasets. (C) Western-blotting assay indicates the expression of TIMELESS is downregulated by interfering with siRNA. (D) U251 sensitivity to SR9009 and SR9011.

**Supplementary Figure 15 |** Overall survival analysis based on high or low riskScore in pan-cancer analysis. ACC: adrenocortical carcinoma (A, P-value< 0.0001), ESCA: esophageal carcinoma (B, P-value = 0.006), KICH: kidney Chromophobe (C, P-value = 0.001), KIRC: kidney renal clear cell carcinoma (D, P-value = 0.003), LIHC: liver hepatocellular carcinoma (E, P-value = 0.022), LUAD: lung adenocarcinoma (F, P-value = 0.001), LUSC: lung squamous cell carcinoma (G, P-value = 0.01), OV: ovarian serous cystadenocarcinoma (H, P-value = 0.002), PAAD: pancreatic adenocarcinoma (I, P-value = 0.007), READ: rectum adenocarcinoma (J, P-value = 0.005), UCEC: uterine corpus endometrial carcinoma (K, P-value = 0.012), UVM: uveal Melanoma (L, P-value = 0.03). No statistical significance of survival analysis of other tumors was observed in the TCGA dataset.
